# Transcriptomic profiling of murine GnRH neurons reveals developmental trajectories linked to human reproduction and infertility

**DOI:** 10.7150/thno.91873

**Published:** 2025-02-26

**Authors:** Yassine Zouaghi, Daniel Alpern, Vincent Gardeux, Julie Russeil, Bart Deplancke, Federico Santoni, Nelly Pitteloud, Andrea Messina

**Affiliations:** 1 Department of Endocrinology, Diabetes and Metabolism, Centre Hospitalier Universitaire Vaudois (CHUV), 1011 Lausanne, Switzerland.; 2 Faculty of Biology and Medicine, University of Lausanne, 1011 Lausanne, Switzerland.; 3 Laboratory of Systems Biology and Genetics, Institute of Bioengineering, School of Life Sciences, Ecole Polytechnique Fédérale de Lausanne (EPFL) and Swiss Institute of Bioinformatics, Lausanne, Switzerland.

**Keywords:** RNAseq, human reproduction, GnRH neuron, embryonic development, genetics

## Abstract

**Rationale:** Neurons producing Gonadotropin-Releasing Hormone (GnRH) are essential for human reproduction and have to migrate from nose to brain during prenatal life. Impaired GnRH neuron biology results in alterations of the reproductive axis, including delayed puberty and infertility, with considerable effects on quality of life and metabolic health. Although various genes have been implicated, the molecular causes of these conditions remain elusive, with most patients lacking a genetic diagnosis.

**Methods:** GnRH neurons and non-GnRH cells were FACS-isolated from mouse embryo microdissections to perform high-resolution transcriptomic profiling during mouse embryonic development. We analyzed our dataset to reveal GnRH neuron molecular identity, gene expression dynamics, and cell-to-cell communication. The spatial context of candidate genes was validated using *in situ* hybridization and spatial transcriptomic analysis. The possible links with human reproduction in health and disease were explored using enrichment analysis on GWAS data and analyzing the genetic burden of patients with congenital GnRH deficiency.

**Results:** GnRH neurons undergo a profound transcriptional shift as they migrate from the nose to the brain and display expression trajectories associating with distinct biological processes, including cell migration, neuronal projections, and synapse formation. We revealed a timely and spatially restricted modulation of signaling pathways involving known and novel molecules, including Semaphorins and Neurexins, respectively. A particular set of genes, whose expression in GnRH neurons timely rises in late developmental stages, showed a strong association with GWAS genes linked with human reproductive onset. Finally, some of the identified trajectories harbor a diagnostic potential for congenital hypogonadism. This is supported by genetic analysis in a large cohort of patients affected by congenital GnRH deficiency, revealing a high mutation burden in patients compared to healthy controls.

**Conclusion:** We charted the landscape of gene expression dynamics underlying murine GnRH neuron embryonic development. Our study highlights new genes in GnRH neuron development and provides novel insights linking those genes with human reproduction.

## Background

Reproductive health encompasses diverse conditions with significant implications for fertility, metabolism, well-being, and aging 1. Central to human reproduction are gonadotropin-releasing hormone (GnRH) neurons, which govern the activity of the hypothalamic-pituitary-gonadal (HPG) axis and, ultimately, the secretion of sex steroids 2. GnRH neurons originate from the nasal placode and migrate from the nose to the hypothalamus during embryonic development 3-6. This peculiar ontogenetic event is conserved in humans and, when defective, leads to Kallmann Syndrome, characterized by the lack of puberty (congenital hypogonadotropic hypogonadism, CHH) and the sense of smell 7, 8. Other defects in GnRH neuron biology have been implicated in several reproductive disorders, including normosmic CHH (nCHH), altered timing of puberty, and polycystic ovary syndrome 9. However, the genetic and molecular mechanisms linking GnRH neuron biology to these disorders remain unclear.

Despite some inter-species differences, the mouse model is a reference for GnRH neuron migration and has been extremely useful in defining three main developmental stages 10, 11. (i) Starting at E10.5, GnRH neurons are continuously generated from the olfactory placode and migrate in chains toward the olfactory bulb (OB) through following the olfactory-vomeronasal nerves (ON/VN) and terminal nerve (TN) bundles 4, 6, 10-12. (ii) Starting at E12.5, the leading GnRH neurons begin crossing the nasal-forebrain junction (cribriform plate) at the level of the olfactory bulb, adapting to the new environment and continuing their journey along the terminal nerve (TN), which turns ventrally toward the hypothalamus. (iii) By E14.5, the first GnRH neurons have reached their final location in the hypothalamus and begin building their network by connecting with hypothalamic and extra-hypothalamic targets, including the median eminence (i.e., the site of neurosecretion), while other neurons continue migrating caudally. (iv) At E18.5, all GnRH neurons have reached their final destination, while neural connectivity continues to develop.

The molecular characterization of GnRH neurons has improved dramatically in the past decades, mainly through the extensive use of mouse genetics and precious insight from human genetics 12-15. However, due to their small number and peculiar spatial distribution, GnRH neurons' precise gene expression dynamics and their drivers remain elusive. Herein, we combined a validated FACS-based method to isolate GnRH neurons with a recently developed high-throughput RNA sequencing approach for low-input samples to map the gene expression changes of GnRH neurons during mouse embryonic development. Further, we investigate how these gene expression dynamics affect GnRH neuron biology and the potential implications for human reproduction.

## Results

### GnRH neurons experience a transcriptional shift from nose to brain

**Figure 1:**
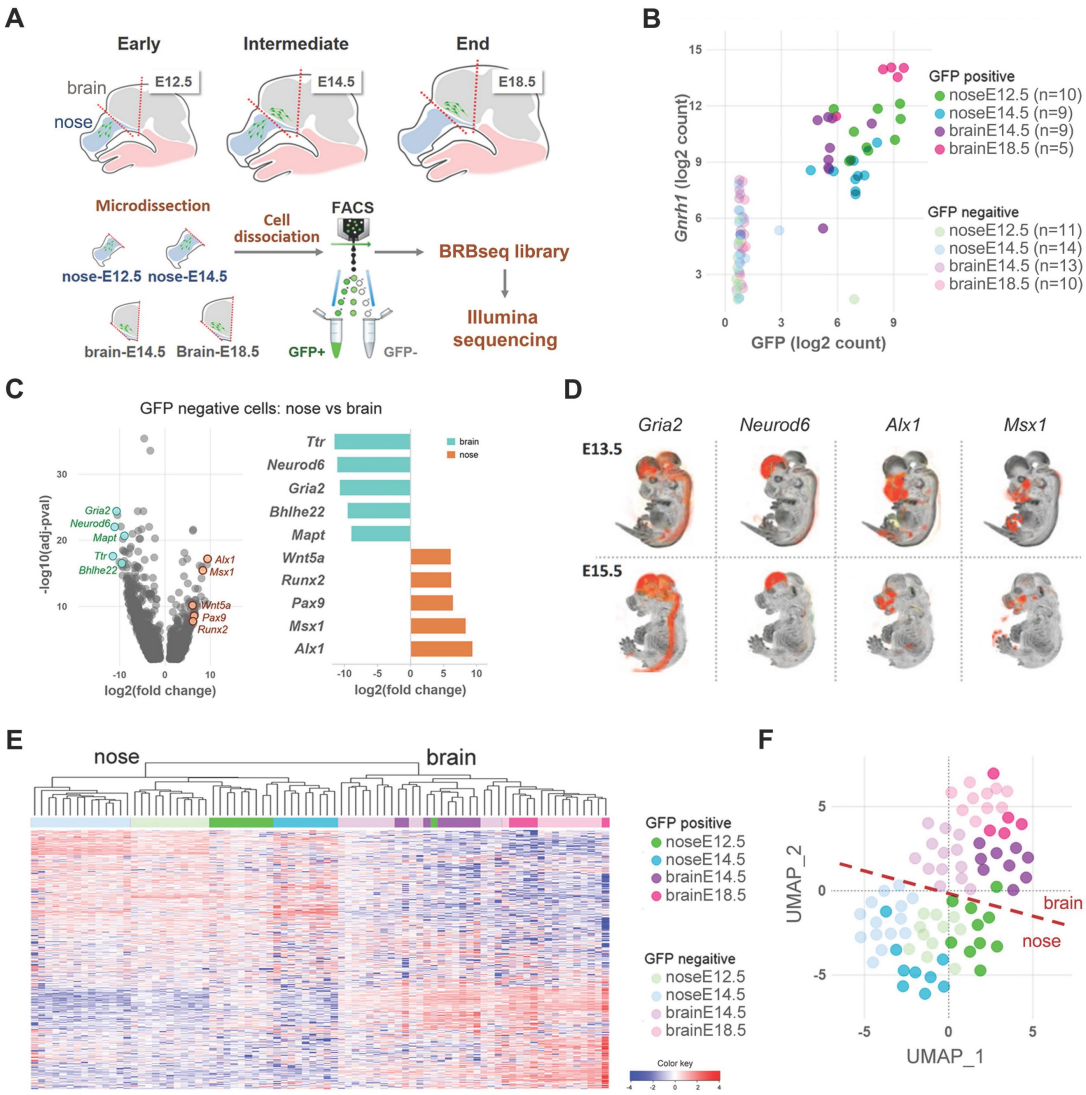
** High resolution profiling of GnRH neurons in the mouse embryo**. (A) Strategy for isolation and RNA sequencing of GnRH+ and GnRH- cells neurons at different developmental stages. Nasal and brain region are microdissected from *GnRH::Gfp* mouse embryos at different developmental stages to follow GnRH neuron migration: early (E12.5 nose), intermediate (E14.5 nose), intermediate (E14.5 brain) and at the end (E18.5 brain). After cell dissociation and FACS isolation, GFP positive and GFP negative cells are processed for BRB library preparation followed by illumina sequencing. (B) Validation of GnRH positive neurons enrichment in the GFP+ fraction by high expression of *Gnrh1* (y axis) and GFP (x axis) transcripts. (C) Differential gene expression analysis of GFP negative cell comparing all nasal (orange) versus all brain (cyan) samples highlights specific regional markers. (D) *In situ* hybridization expression levels of nose and brain regional markers (source: Allen Brain Atlas). (E) Correlation heatmap and hierarchical clustering (complete linkage) of the 1000 most variable genes. (F) Representative dimensional reduction analysis (UMAP).

To depict GnRH neuron expression dynamics, we used a low-input-optimized RNA sequencing approach on FACS-isolated GFP^+^ and GFP^-^ cells from *GnRH::GFP* transgenic embryos at four key spacetime points (Figure 1A). *1) E12.5 nose (cell birth and active migration)*: 100% of GnRH neurons are in the nose/OB while moving caudally toward the brain. *2) E14.5 nose (active migration)*: about 30% of the GnRH neurons are still migrating throughout the nose. *3) E14.5 brain (active migration and axonogenesis/extension)*: the remaining 70% of GnRH neurons have already entered the brain and turned ventrally toward the hypothalamus. *4) E18.5 brain (axonogenesis/extension, synaptogenesis)*: GnRH neuron migration to the brain is completed. GFP^-^ cells, derived from the same anatomical regions during dissection, were included to provide a reference for identifying GnRH specific expression patterns and potential signaling interactions relevant to GnRH neuron development. Their inclusion offers insights into the cellular environment surrounding GnRH neurons. The identity of the sorted cells was confirmed *in silico* by measuring *Gnrh1* transcript relative to GFP expression. Notably, *Gnrh1* transcript was expressed within GFP negative samples, albeit in a low range (Figure 1B), indicating that a small fraction of GnRH neurons might not express the GFP reporter, as previously shown in transgenic reporters 16, 17. The *bonafide* location of GnRH neurons (i.e., microdissection specificity) was confirmed by the overlap of the most differentially expressed genes in all GFP negative samples with specific markers of developing nose and brain tissues (Figure 1C, D).

Surprisingly, cell localization (i.e., nose vs. brain) was the main driver of the first branching in the sample tree and the primary source of variation using hierarchical clustering on the 1000 most variable genes. Cell type (i.e., GnRH^+^ vs. GnRH^-^ cells) occurred at a lower hierarchical level (Figure 1E). Consistently, dimensional reduction 18 and principal component analysis (PCA) confirmed that nose and brain GnRH neurons clustered in two sub-populations (Figure 1F, Figure S1).

### Molecular identity of GnRH neurons

**Figure 2:**
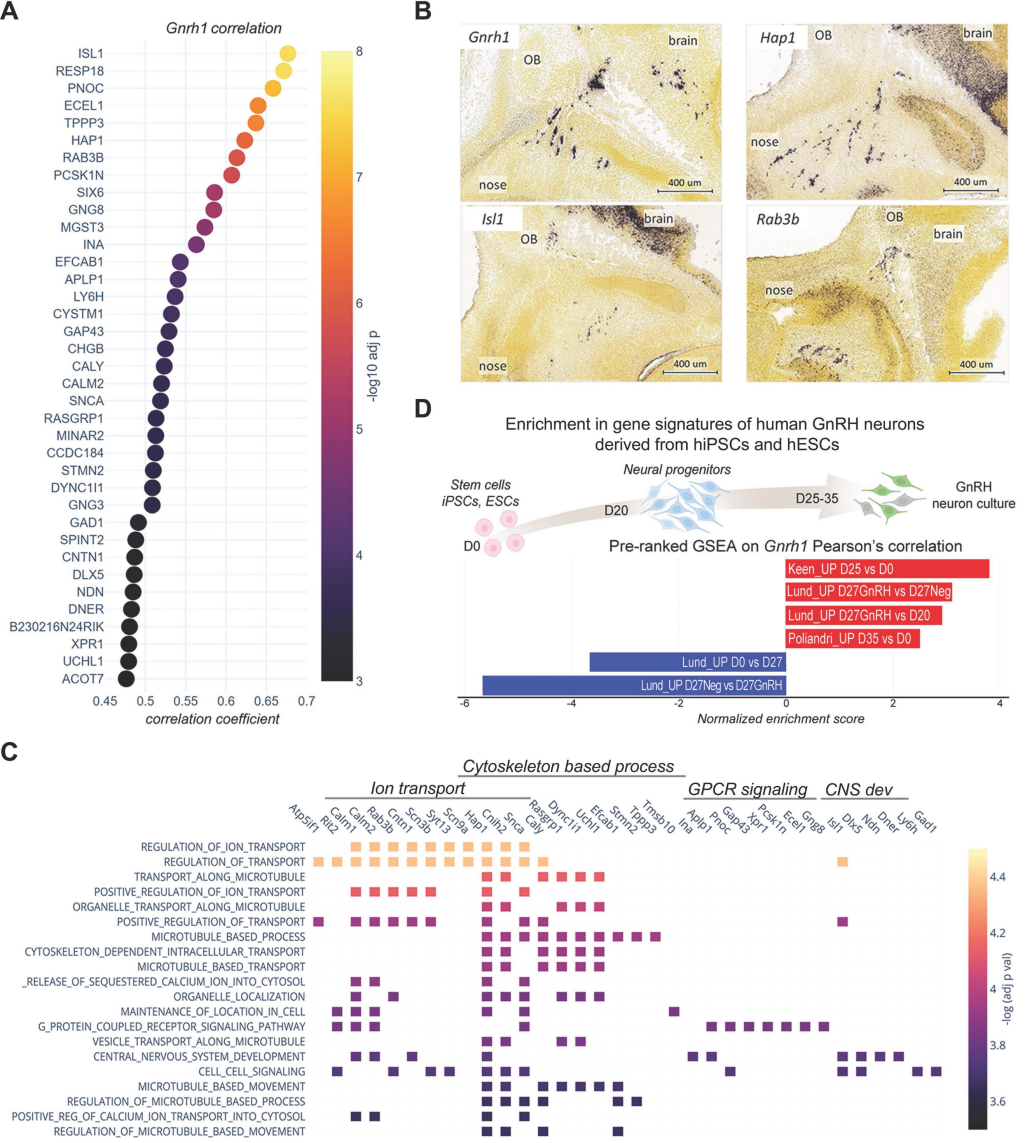
** GnRH neuron molecular identity**. (A) Top 50 genes correlating with *Gnrh1* ranked by correlation coefficient. The x-axis represents the correlation coefficient, while correlation significance is represented by color according to the color bar. Trajectories below the threshold of significance are shown to illustrate p value distribution and colored in yellow. (B) Representative *In situ* hybridization of *Gnrh1* and top correlating genes illustrating their similar expression patterns in sagittal sections from mouse embryos at intermediate developmental stages (E13.5-E15.5). OB, olfactory bulb. Data source: Allen Brain Atlas. (C) Gene sets overlap of the top 50 signature genes with biological functions from the MSigDB database. CNS dev, central nervous system development. Enrichment significance is represented by color according to the color bar. (D) Similarity between mouse and human GnRH neuron molecular profiles is illustrated by significant enrichment of genes correlating with *Gnrh1* within transcriptomic signatures of human GnRH neurons differentiating from human iPSCs; Keen_UP D25 vs D0 (genes up-regulated in GnRH neuron cultures after 25 days of differentiation *in vitro* vs. iPSCs from *Keen et al 2021*); Lund_UP D27GnRH vs D27Neg (genes up-regulated in GnRH neuron cultures after 27 days of differentiation *in vitro* vs GnHR negative cells from *Lund et al., 2020*); Lund_UP D27GnRH vs D27Neg (genes up-regulated in GnRH neuron cultures after 27 days of differentiation *in vitro* vs immature cultures at day 20 from *Lund et al., 2020*); Poliandri_UP D35 vs D0 (genes up-regulated in GnRH neuron cultures after 35 days of differentiation *in vitro* vs. ESC from *Poliandri et al., 2017*).

Current understanding of the molecular features of GnRH neurons remains limited, with Gnrh1 standing as the sole marker reliably distinguishing this population (i.e., consistently expressed in all GnRH neurons while being absent from neighboring cells) 19. To identify genes potentially describing GnRH neuron molecular identity during embryonic development, we used all GFP+ and GFP- samples in the dataset across all ages and regions and ranked all detected genes according to their correlation with *Gnrh1*. Among the top 50 correlated genes (Figure 2A, Table S1), we identified transcription factors essential for GnRH expression, such as *Dlx5* and *Six6* 20-22. *In situ* hybridization of these genes revealed expression profiles following the expected GnRH pattern in the mouse embryo, with some staining in other structures, including the olfactory epithelia and the forebrain (Figure 2B). Notably, the top-ranked gene on our list was *Isl1*, a transcription factor recently identified in developing GnRH neurons in mice and humans 23.

Our analysis identified that most top-ranked genes correlating with Gnrh1 overlap with functional gene sets associated with microtubule-based function, ion transport, and GPCR signaling (Figure 2C). While these biological processes are not unique to GnRH neurons, their enrichment reflects key developmental processes active during GnRH neuron migration and differentiation. The strong correlation of these genes with Gnrh1 suggests that they represent molecular features of GnRH neurons' transcriptional state, particularly during their migration and axonogenesis. *Stmn2* and *Stmn3* encode for Stathmins, microtubules-destabilizing proteins previously implicated with GnRH neuron migration 24. *Gap43*, encoding for the growth-associated protein 43, is expressed in developing olfactory and GnRH systems, although its biological role is not yet elucidated 25, 26. *Dync1i1* encodes for a subunit of a microtubule-based motor protein, which is involved with split-hand-foot-malformation 27, 28 a known CHH-associated phenotype 29. Other genes are involved in ion transport and encode voltage-gated sodium channels (*Snc3b* and *Scn9a*), but also proteins modulating the activity of voltage-gated calcium channels (*Cbarp*) or controlling ion channel localizations (*Hap1*).

One central question related to the molecular identity of GnRH neurons is its conservation across species. To investigate the mouse-human similarity, we used available RNAseq data from recent studies describing the *in vitro* differentiation of GnRH neurons from human ESCs and iPSCs 30-32. Indeed, gene set enrichment analysis in our data revealed a positive association of the mouse genes correlating with *Gnrh1* with gene signatures of human GnRH neurons (Figure 2D).

Altogether, these findings indicate that the molecular identity of GnRH neurons is complex and may involve a diverse set of transcription factors and other regulatory genes beyond *Gnrh1*.

### Spatiotemporal expression trajectories define GnRH neuron developmental stages

**Figure 3:**
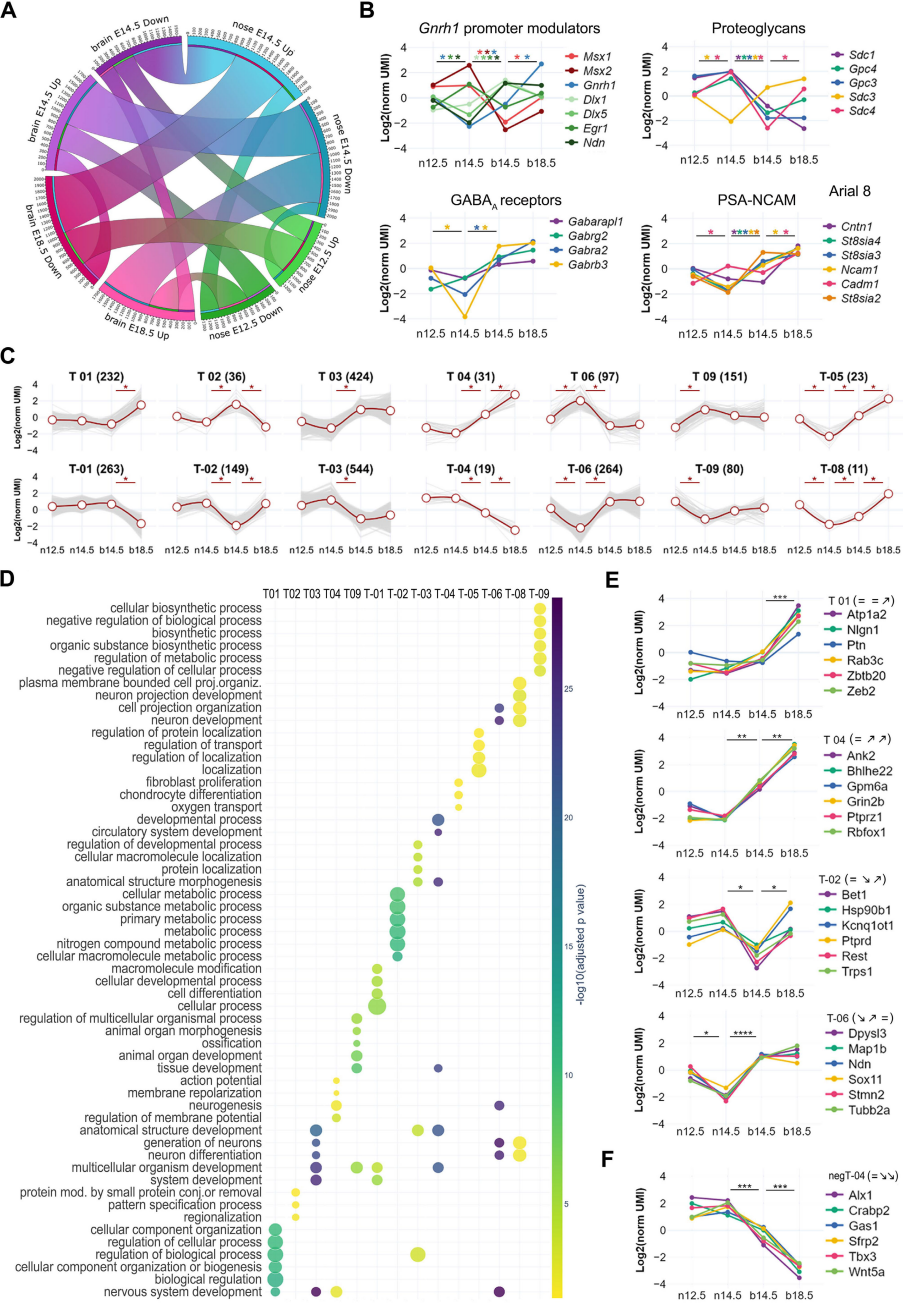
** Spatio-temporal trajectories delineate GnRH neuron expression dynamics**. (A) Chord diagram representing the proportion of genes with statistically significant up- or down-regulation in GnRH neurons across different conditions. Developmental stages are represented by nodes in different colors while the number of significant genes between two conditions is proportional to the thickness of the lines connecting the nodes. (B) Normalized expression profiles and trajectory classification of known genes involved in GnRH neuron development, including modulators of *Gnrh1* promoter, proteoglycans, GABA_A_ receptors, and genes involved in the formation of Polysialic Acid Neural Cell Adhesion Molecule (PSA-NCAM). (C) Expression dynamics of the main GnRH neuron gene trajectories (i.e. at least 10 genes per trajectory). Average gene expression is shown in red while light grey lines represent individual genes. Asterisks indicate significant gene expression changes across consecutive developmental stages. (D) Scatter plot showing the top six significant terms in the main trajectories after functional enrichment analysis within biological processes from gene ontology database (see detailed statistical report In Table S4) Enrichment significance is represented by color according to the color bar. E,F) Normalized expression profiles and trajectory classification of top genes emerging from representative trajectories significant after functional enrichment analysis. Asterisks indicate significant gene expression changes across consecutive developmental stages (see detailed statistical report In Table S2 and S3).

Using transcriptome analysis of GnRH-positive neurons, we identified wide transcriptional dynamics across different developmental stages (Figure 3A-C). We employed a step-wise differential expression analysis across consecutive developmental stages to simplify this complexity and classify genes into spatiotemporal trajectories (Figure S2, S3, Table S2 and Methods). These trajectories parallel known biological processes occurring in GnRH neurons across development, such as the timely increase of Necdin 12 and DLX transcriptional activators and decrease of MSX repressors modulating *Gnrh1* expression 20 (Figure 3B). Other examples are the "early stages trajectories" of genes encoding for syndecans (e.g., *Sdc1* and *Sdc4*), glypicans (e.g., *Gpc3* and *Gpc4*) which promote GnRH neuron migration 12, 33-35, and the "brain trajectories" of genes involved in late stages, including genes encoding GABA receptor subunits 36, 37 (e.g., *Gabrb3* and *Gabrg2*) and other proteins critical for the establishment of functional networks such as PSA-NCAM (*Ncam1*, St8sia3), SynCAM (*Cadm1*) and contactin (*Cntn1*) 38-40 (Figure 3B).

Next, we combined spatiotemporal trajectories with functional enrichment analysis using GnRH trajectories as multi-query input to identify spatially and temporally restricted biological processes throughout GnRH neuron development. Distinct biological processes were preferentially associated with trajectories up-regulated at particular developmental stages, including cell migration (GO:0016477) during the migration from nose to brain (T-03, p = 1.28E-17) and regulation of neuron projection development (GO:0010975) at late developmental stages (T03, p = 1.90E-19) (Figure 3C, D, Figure S4 and Table S4). These associations show little overlap across trajectories and are consistent with the idea that specific gene trajectories may drive spatiotemporally restricted biological functions to shape GnRH neuron development. Next, we selected the most differentially expressed genes associated with previously identified top GO terms (Figure 3E, Figure S5). Some genes were already linked with GnRH neuron biology or reproductive phenotype. Indeed, consistent with their trajectories, the microtubule-associated protein stathmin (*Stmn2*) and the repressor element-1 silencing transcription factor (*Rest*) promote migration in immortalized GnRH neurons 24, 41.

The trajectory analysis of GnRH-negative cells (Table S3) revealed T-box transcription factor 3 (*Tbx3*) as a gene with high expression in the early stages in the nasal compartment (Figure 3F). Notably, its human orthologue is mutated in patients with ulnary mammary syndrome (OMIM# 181450), a condition associated with CHH 42. Another candidate in the same trajectory is a member of the Wnt family (*Wnt5a*). This expression profile is consistent with previous detection in the olfactory mesenchyme 43 and its ability to activate olfactory ensheathing cells 44, a population of migrating glial cells providing a permissive microenvironment and guidance for GnRH neuron migration 45.

Candidates up-regulated in GnRH neurons at the end of migration are of particular interest due to their potential contribution to GnRH neuron maturation and establishment of their connections with key neuro-glial partners which is critical for the onset of puberty and fertility homeostasis. We found *Nlgn1* (T 01), which belongs to the neurexins and neuroligins family, a group of cell-cell adhesion molecules involved in synaptic formation and plasticity 46. Notably, *Nlgn3,* another member of this family, has a similar trajectory (T 03), and mutations in its human orthologue have been identified in CHH patients 47. Another appealing candidate with increased expression at the end of migration (T 01) is Pleiotrophin (*Ptn*), a secreted heparin-binding growth factor that binds to the perineuronal net (PPNs) 48. Pleiotrophin binds with high-affinity Posphacan (*Ptprz1*) and has been shown to modulate migration and induce neurite outgrowth 48-50 (Figure 3E). Notably, several *Ptprz1* paralogs (*Ptprs*, *Ptprd*, *Ptpro*, *Ptprf*) are dynamically expressed in GnRH neurons and, together with the other member of their family, have been implicated with the timing of puberty through genome-wide association studies (GWAS) 51.

Altogether, these findings show that complex expression dynamics are a distinctive feature of genes controlling GnRH neuron development. These genes drive the timely activation of key biological processes and can be identified by spatiotemporal trajectory analysis.

### Dynamic networks of cell-to-cell communication highlight timely signaling pathways for GnRH neuron development

Based on the extent of the observed transcriptional dynamics, we anticipated that the cell-to-cell-communication (CCC) between GnRH neurons and their surrounding environment could be affected by expression changes in specific ligands (environment) and receptors (GnRH neurons). CCC analysis has recently improved thanks to the growing availability of accurate protein-protein interaction (PPI) databases and is now widely used to construct tissue and cell-specific ligand-receptor networks by inferring active signaling pathways based on synchronized expression from transcriptomic data 52.

**Figure 4:**
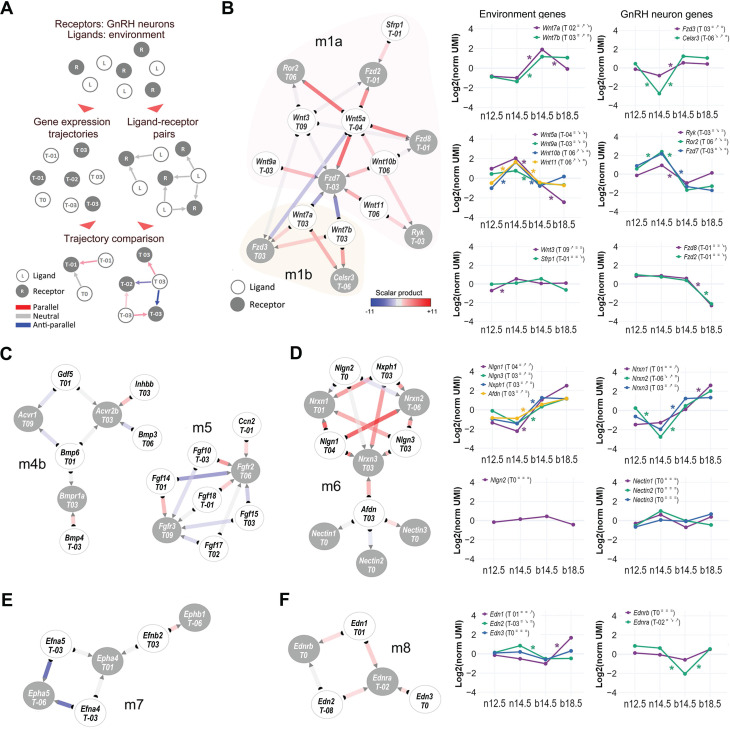
** Dynamic cell-to-cell communication networks in GnRH neuron development.** (A) Schematic illustrating the strategy to infer dynamic interactions between GnRH neurons and their environment. Ligand-receptor interaction data are combined with gene expression trajectories (see Methods). Edge color represents the inferred ligand-receptor interactions, edge colors represent the directionality of LR expression trends (calculated as the scalar product of gene expression): parallel (ligand and receptor increase/decrease together), anti-parallel (ligand and receptor show opposing trends), or neutral (no relationship). The inferred PPI network and gene expression profiles of different modules of ligands and their respective receptors including (B) Wnt submodules m1a and m1b, (C) BMP submodules m4b and FGF module m5, (D) Neurexins and Neuroligins in module m6, (E) ephrins module m7, and (F) endothelin module m8. Node color discriminate between receptors expressed in GnRH neurons (grey) and ligands expressed in the environment (white). The degree of coordinated expression (i.e., scalar product) is color-coded on edges. Color-coded asterisks indicate significant gene expression changes across consecutive developmental stages (see detailed statistical report In Table S2 and S3).

We combined a curated database of ligand-receptor interactions (CellTalkDB) 53 with our trajectory analyses on GnRH neurons and GnRH-negative cells to construct a dynamic protein-protein interaction network of directional paracrine communication between the environment and GnRH neurons. Gene trajectories were annotated on ligand and receptor nodes, while the strength of ligand-receptor interactions was calculated based on the expression correlation of ligands and receptors in each pair (Figure 4A).

Based on the local connectivity and the inferred strength of ligand-receptor pairs, we used the resulting network to identify different functional modules and sub-modules. As expected, many of those contained gene families and pathways already linked with GnRH neuron development, such as semaphorin family members and their receptors, plexins, and neuropilins (Figure S6, module m3). Semaphorins play a crucial role in brain development and GnRH neuron development, with different members of this signaling pathway eliciting opposing effects depending on the molecular environment 54-56. Other modules display elements of the Wnt (m1), BMP (m4b), and FGF (m5) signaling pathways (Figure 4B, C), which are well known for their cooperative role in the patterning of the olfactory placode and its neurogenic niche 57. Consistent with these functions, we found one of the two Wnt submodules (m1a: *Wnt5a, Wnt9a, Wnt10b, Wnt11, Ryk, Ror2, Fzd7*) with an early stages activation profile. However, the second submodule has an opposite spatiotemporal profile (m1b: *Wnt7a, Wnt7b, Fzd3, Celsr3*), suggesting that some components of Wnt signaling might be involved in later events, including GnRH neuron maturation and connectivity. Our analysis highlights pathways previously linked with mouse GnRH neuron development in mice and humans like DCC/Netrin (m9) 58-60 and strengthens old candidates such as ephrins (m7) 61 and endothelins (m8) 62, 63 (Figure 4E-F).

One of the most compelling findings of this analysis is the identification of the neurexins/neuroligins module (m6) presenting with highly coordinated expression at the end of the migratory phase (Figure 4D). Neurexins are presynaptic transmembrane proteins that interact with postsynaptic neuroligins to form trans-synaptic complexes, which play a critical role in regulating synaptic function and facilitating synapse formation and maintenance and could be involved in the establishment of GnRH neuron connections with other neurons 46, 64, 65.

### Spatial validation of candidate CCC-ligands and markers for GnRH neuron development

**Figure 5:**
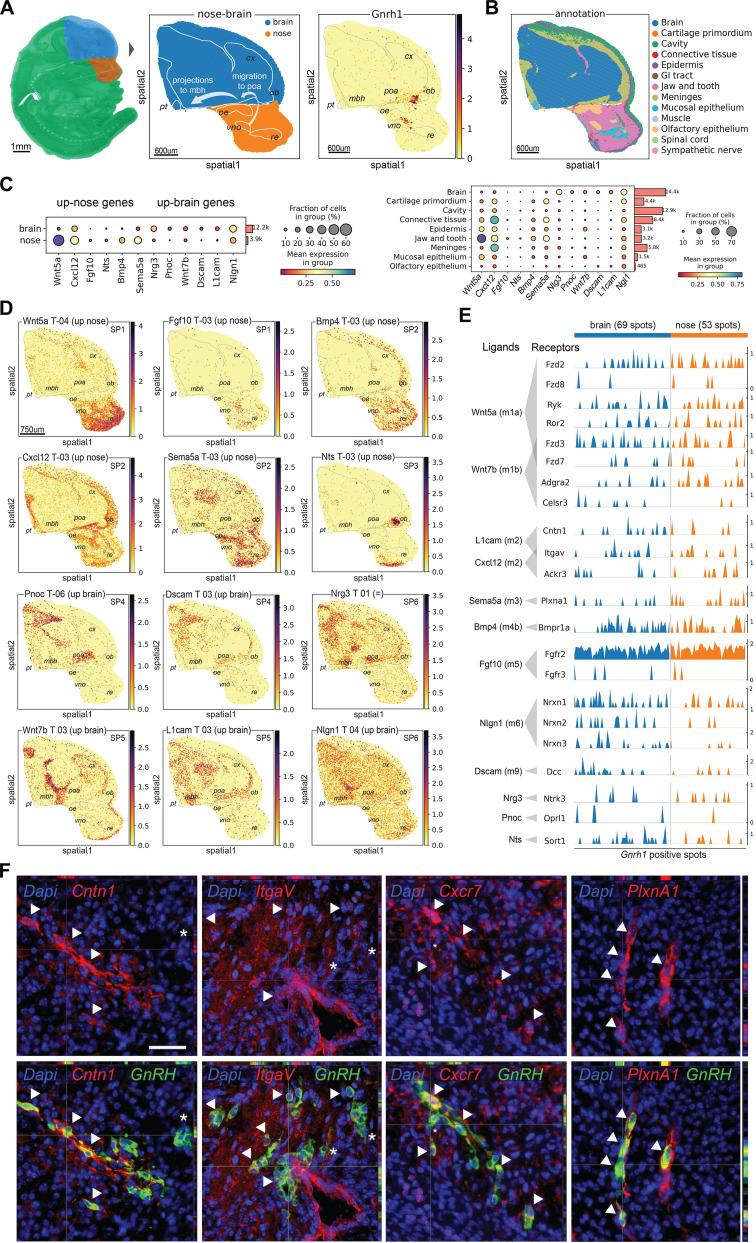
S**patial transcriptomics validation of candidate genes for GnRH neuron development.** (A) Embedding plots showing spatial visualization of transcriptomic data reanalyzed from a mouse embryo section at E13.5 (MOSTA). (Left) selection of the nose and forebrain as regions of interest. (Middle) annotation of anatomical regions relevant for GnRH neuron development. Bins color represents region annotation. (Right) spatial visualization of *Gnrh1* transcript expression in the context of relevant anatomical regions (gray dotted lines). (B) Regional annotation based on local gene expression from MOSTA original publication. (C) Dot plots illustrating the spatial transcriptomic expression of representative ligand-encoding genes up-regulated in the nose or brain, as identified in our bulk RNAseq analysis (Figure 4, Figure S3). The left panel compares gene expression between nose and brain regional annotations (as per our bulk analysis), while the right panel identifies local sources by quantifying expression across different tissue components. Dot size represents the fraction of cells expressing a gene in each group, and color indicates the average gene expression level in each group. (D) Embedding plots showing spatial expression of representative ligand-encoding genes illustrating regional expression (nose vs brain) as predicted by our expression trajectory analysis based on bulk RNAseq of mouse head microdissections. Specific spatial patterns (SP) relative to anatomical regions relevant for GnRH neuron development are also indicated. (E) Trak plot showing the expression of receptor-encoding genes in individual *Gnrh1*^ +^ spots as vertical lines grouped by region annotation (nose vs brain). Gene expression levels are represented by the heights of vertical lines. (F) Representative double immunofluorescences on E14.5 mouse sagittal sections illustrating heterogeneous expression of Contactin1 (*Cntn1*), Integrin alpha V (*Itgav1*), C-X-C chemokine receptor type 7 (*Ackr3*) and PlexinA1 (*PlxnA1*) in migrating GnRH neurons. Double positive cells are indicated by arrowheads while asterisks depict neurons expressing only GnRH. Scale bar 50 um.

To better understand how genes involved in communication with the local environment may contribute to GnRH neuron development, we performed spatial transcriptomic validation using MOSTA, a Mouse Organogenesis Spatiotemporal Transcriptomic Atlas with 53 sagittal embryo sections from E9.5 to E16.5 66. After extracting data from sections containing the Gnrh1 transcript, we annotated anatomical structures and regions associated with GnRH neuron development, mapping gene expression patterns along the GnRH neuron migratory route with near-cell resolution (Figure 5A).

Spatial profiles of CCC-ligand genes indicate preferential localization in the nose or the brain, according to our expression trajectory classification (e.g., *Wnt5a*: up-nose, *Nrg3*: up-brain) (Figure 5A-C and Figure S7 and S8). Using detailed anatomical annotations, we identified six major spatial patterns linking CCC-ligand genes to distinct steps of GnRH neuron development. The first pattern (**SP1**) is characterized by genes expressed around the start site of GnRH neuron migration, which is located in the developing vomeronasal/olfactory epithelium (Figure 5D). SP1 genes are primarily found in the nasal mesenchyme surrounding the VNO and show a gradient towards the tip of the nose (e.g., *Wnt5a*) or between the VNO and the OB along the GnRH neuron migratory route toward the brain (e.g., *Fgf10*). The second spatial pattern (**SP2**) includes genes expressed along the GnRH neuron migratory route. These genes are detected both in the nasal mesenchyme between VNO and the OB and in mesenchymal tissue outside the nose corresponding to the most ventral part of the developing meninges (e.g., *Bmp4*, *Cxcl12*, *Sema5a*).

**SP3** genes (e.g., *Nts*) follow a more specific pattern with their expression related to the nasal/forebrain junction. In this region, which includes the ventral side of the OB, GnRH neurons enter the brain before migrating towards the preoptic area of the hypothalamus, while a subset of cells follows an alternative path and migrates around the OB 11. Considering the CCC-ligands with higher expression in the brain, we identified three spatial patterns linked with the preoptic area of the hypothalamus (POA) and the ventral part of the mediobasal hypothalamus (MBH), two regions representing the main targets of GnRH neurons in terms of migration and neuronal projections, respectively 12, 13. In particular, we found genes mainly expressed in the POA (e.g., Pnoc, Dscam; SP4), in the ventral part of the MBH (e.g., *Wnt7b*, *L1cam*; SP5), and genes equally expressed in both regions (e.g., *Nrg3*, *Nlgn1*; **SP6**) (Figure 5E, Figure S8).

Further, we investigated whether spatial transcriptomics would be useful for validating gene expression within GnRH neurons. Our analysis confirmed various degrees of co-expression with *Gnrh1* transcript for several CCC-receptor genes in both nasal and brain regions (Figure 5E and Figure S9). Due to the limited number of GnRH neurons, a quantitative assessment of gene expression differences between nose and brain GnRH neurons was not possible. However, we observed that CCC-receptors are not expressed in all GnRH neurons at the same level. Among the genes heterogeneously expressed, we identified some encoding receptors for the same ligand molecules, such as *Cntn1*, *Itgav*, and *Ackr3*, expressed in spatially restricted spots containing GnRH neurons (Figure S10). To spatially validate these results beyond the near-cell resolution of our spatial transcriptomic approach (i.e., spot size, 25 µm), we performed double immunostaining followed by fluorescence microscopy analysis (Figure 5F). These experiments confirmed the colocalization of selected receptor-encoding genes with GnRH neurons providing cellular-level evidence that complements the spatial transcriptomic data.

**Figure 6:**
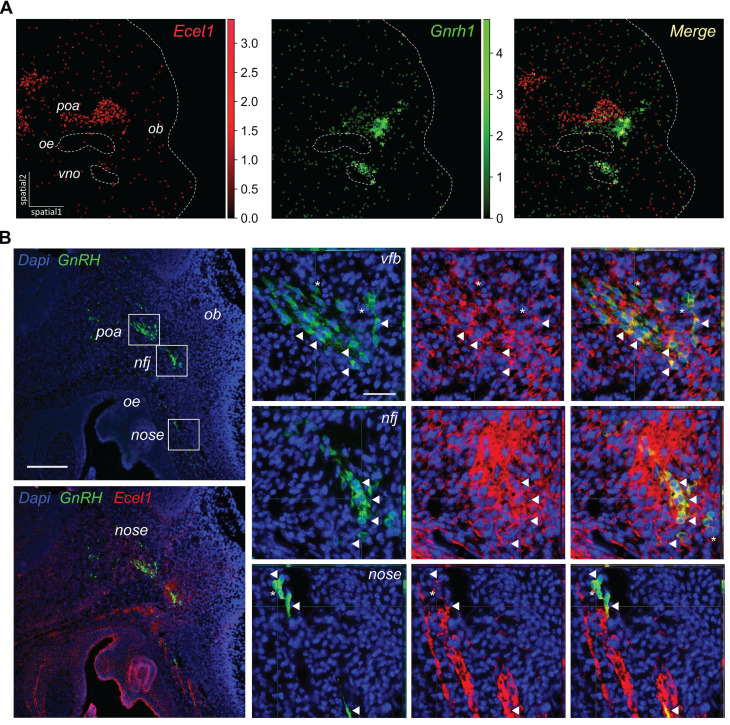
** Spatial transcriptomics validation of candidate markers for GnRH neurons.** (A) Embedding plots of transcriptomic data reanalyzed from a mouse embryo section at E13.5 (MOSTA) illustrating the co-localisation of the candidate marker *Ecel1* with *Gnrh1* transcript. Colored scalebars represent relative gene expression intensity. Section border and relevant anatomical regions are outlined (light gray dotted lines). Spot size 25um. (B) Representative double immunofluorescences on E14.5 mouse sagittal sections illustrating the expression of Ecel1 in GnRH neurons at different sites along their migratory route. Double positive cells are indicated by arrowheads while asterisks depict neurons expressing only GnRH. Scale bar 50 um. *poa, preoptic area of the hypothalamus; ob, olfactory bulb; nfj, nasal-forebrain-junction oe, olfactory epithelium; vno, vomeronasal organ*.

Finally, we sought to use spatial transcriptomics to cross-validate the candidate markers for GnRH neurons identified in our RNA-seq analysis. First, we used *Gnrh1* transcript within the spatial dataset to pinpoint GnRH neurons and calculate a spatial specificity coefficient for candidate GnRH neuron markers (Table S5). As expected, the top five markers taken individually label many of the spots positive for *Gnrh1* transcripts, with overall good spatial specificity and limited expression in other structures (e.g., OE, VNO and POA) (Figure 6A, Figure S11). However, none of the marker genes used individually was able to label all GnRH neurons, suggesting that these genes are heterogeneity expressed within GnRH neurons. In contrast, when we combined the top marker genes by collapsing their expression into modules containing different number of top genes, all GnRH neurons were labeled with increasing specificity (Figure S12). Interestingly, double immunofluorescence analysis indicated that while relatively specific, these markers are not exclusive to GnRH neurons (Figure 6B). For example, Ecel1, which appeared highly specific in the nasal region, was also found to be expressed in cell clusters that seem to migrate alongside GnRH neurons in the nasal region. Additionally, in the preoptic area (POA), Ecel1 was observed to be less specific, as it appears to be expressed by other neuropeptidergic neurons, reflecting regional variability in marker specificity.

### GnRH neuron trajectories are linked with the genetics of human reproduction

Combining spatiotemporal trajectories with PPI, we identified genes critical for GnRH neuron biology in rodents and genes previously linked with human reproduction, including *NLGN3*, *DCC*, *NTN1*, and members of the PTPR, Semaphorin/Plexin, and Neuropilin families. These findings prompted us to investigate whether expression trajectories could reveal genetic association with relevant aspects of human reproduction in the general population and patients with congenital GnRH deficiency (CHH).

To assess the genetic association between Gnrh neuron expression trajectories, we performed large-scale genome-wide enrichment analysis, a well-established approach to link biological processes to specific traits through significant GWAS signals 67-69. We first used the GWAS catalog to retrieve a list of genes associated with human traits linked with reproductive onset (i.e., age at menarche, EFO_0004703; age at menopause, EFO_0004704; age at the first sexual intercourse, EFO_0009749). (Table S6). We used these genes to test their enrichment against the GnRH neuron trajectories (Figure 5A) and found T01 as the top-ranked trajectory (P_adj_=5.8x10^-5^), followed by T03 (P_adj_=2.1x10^-2^). Notably, T01 trajectory contains genes up-regulated at the end of GnRH neuron migration, controlling neuronal projections and synaptic contacts' formation (Table S2 and S4). In other words, genetic variability in genes belonging to trajectory T01 may explain, at least in part, differences in the age of reproductive onset in the normal population, suggesting that trajectory classification could highlight sets of genes with a higher probability of being mutated in congenital GnRH deficiency.

**Figure 7:**
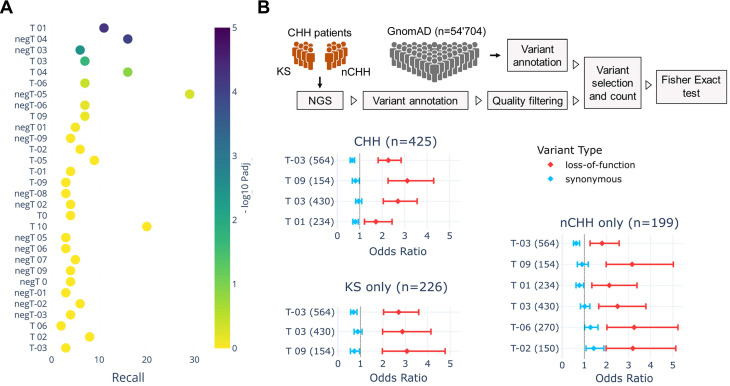
** Genetic overlaps linking GnRH neuron trajectories with human reproduction.** (A) Scatter plot illustrating the enrichment across GnRH neuron trajectories of GWAS genes associated with reproductive onset (i.e., puberty, age at menarche, age at first sexual intercourse, and age at menopause from GWAS catalog). The x-axis represents the recall, calculated as the ratio between intersection size and term size, while enrichment significance is represented by color according to the color bar. Trajectories below the threshold of significance are shown to illustrate p value distribution and colored in yellow. (B) Forest plots showing the mutation load of rare PTVs relative to synonymous in patients with CHH, KS, and nCHH vs GnomAD controls. The x axis represents the odds ratio which quantifies the likelihood of observing mutations in a gene trajectory in cases compared to controls. Error bars represent the 95% confidence interval of the odds ratio. Only trajectories with significant burden in cases vs control are shown. Detailed statistical report available In Table S7, S8 and S9. The full list trajectory genes see Table S2.

To test this hypothesis, we used a gene-set burden analysis in cases vs controls, a population-based genetic analysis extensively used to associate traits or diseases with genes grouped in biologically relevant gene sets 70-72. This approach helps to compensate for the low sample size of rare disease cohorts by measuring the cumulative effects of rare variants instead of analyzing one variant at a time. We focused on rare protein-truncating variants (PTVs) relative to synonymous by performing a trajectory-collapsed burden test in CHH patients (n=425) vs GnomAD controls (Table S7). Four trajectories showed significant enrichment: T-03 (P_adj_=2.3x10^-18^), T09 (P_adj_=1.9x10^-11^), T03 (P_adj_=3.7x10^-10^), and T01 (P_adj_=1.0x10^-3^) (Figure 7B). Knowing the different genetic architecture of CHH relative to specific sub-phenotypes, we repeated the analysis separating KS and normosmic CHH (nCHH) diagnoses, which are preferentially associated with developmental and homeostatic pathogenic mechanisms 73, 74. In line with the hypothesis, the most significant enrichments were driven by PTVs found in KS patients, including T-03 (KS P_adj_=2.1x10^-13^ nCHH P_adj_=10^-5^) and T03 (KS P_adj_=2.9x10^-7^ vs. nCHH P_adj_=1.6x10^-3^) (Figure 7B Table S8 and S9). On the other hand, some trajectories appeared significant exclusively in nCHH patients, T01 (P_adj_=1.3x10^-3^), T-06 (P_adj_=6.1x10^-3^), and T-02 (P_adj_=4.6x10^-2^).

Altogether, these results show that different GnRH neuron trajectories align with distinct aspects of human reproduction, particularly genes up-regulated in late embryonic development with age at puberty and early genes with Kallman Syndrome.

In-depth analysis of individual candidate genes, including phenotype-genotype correlation analysis and variant functional validation, is out of the scope of this study. However, these results show that GnRH neuron trajectories are linked with human genetic determinants driving physiological and pathological variability in human reproduction.

## Discussion

Our study provides high-resolution transcriptomic profiling of GnRH neurons during migration in the mouse embryo. By analyzing more than 20'000 GnRH^+^ cells from the mouse embryo, this work delineates the rich expression dynamics of GnRH neurons along four pseudo-timepoints. This work expands the scope of previous studies 75-78 and enhances our understanding of the molecular mechanisms underlying GnRH neuron ontogeny.

We identified a profound transcriptional shift from the nose to the brain when GnRH cells drastically change their environment. This shift transcends the variability between cell types (GnRH^+^ and GnRH^-^ cells) and involves the upregulation of genes shaping neuronal activity including encoding voltage-gated sodium channels (e.g., *Snc3b* and *Scn9a*)*,* glutamate ionotropic receptor subunits (e.g., *Grina* and *Grin2b*), and GABA receptors (e.g., *Gabra2* and *Gabrb3*). These results are in line with previous observations showing a role of electrical activity modulators in GnRH neurons migration, with a main role of GABA receptors 77, 79-82. Moreover, this data is reminiscent of a recent study in zebrafish larvae showing a pause of the migration of the GnRH cells at the nasal-forebrain junction (NFJ), during which they acquire coordinated neuronal activity required to enter the brain 83.

One limitation in the study of GnRH neurons is the reliance on Gnrh1 as the sole marker for their identification. While Gnrh1 is the canonical marker, its expression can vary at the transcriptional and post-transcriptional levels, which may lead to underrepresentation or misidentification of GnRH neurons. This highlights the importance of identifying alternative markers for a more accurate and comprehensive characterization of GnRH neurons. Our analysis identified several high-ranked genes correlating with *Gnrh1* across development, which could help define GnRH neuron identity. The top-ranked gene was ISL LIM homeobox 1 (*Isl1*), a transcription factor initially identified in pancreatic islet cells 84 and known for controlling migration, axonal pathfinding, and maturation of peripheral neurons 85-87. Expression of *Isl1* was recently found in specific neuronal lineages originating from the olfactory placode, including GnRH neurons in birds, mice, and human fetuses, as well as in GnRH neurons derived from human induced pluripotent stem cells (iPSC) 23, 31, 88. Being expressed both in GnRH progenitors and GnRH neurons, Isl1 has been proposed as a candidate gene for GnRH neuron development. However, conditional deletion in postmitotic GnRH neurons didn't result in evident impairment of GnRH neuron migration or GnRH expression, probably due to low knockout efficiency or compensatory mechanisms 23, 88. Further studies are needed to establish the functional role of this gene in the prenatal development of GnRH neurons.

Genes highly co-expressed with *Gnrh1* appear involved in specific biological processes, although not unique to GnRH neurons (i.e., cytoskeleton remodeling and ion transport). This evidence aligns with our *in situ* validation based on *ISH* and spatial transcriptomics analysis. Indeed, when considered individually, we found that these potential markers label GnRH neurons with heterogeneity at single-cell scale and varying degrees of spatial specificity. However, combining top markers genes into modules allowed us to spatially resolve GnRH neurons independently on *Gnrh1* transcript. Gene expression heterogeneity in GnRH neurons aligns with previous observations and is supported by our double immunofluorescence validation of CCC receptors (e.g., Cntn1, Itgav, and Ackr3), which exhibit spatially restricted expression patterns within GnRH neurons. Further studies integrating single-cell analysis and functional experiments are needed to better characterize GnRH neurons' molecular identity and to understand the functional consequences of their expression heterogeneity across development.

Resolving gene expression dynamics based on spatiotemporal trajectories of GnRH neurons highlights specific biological processes at different developmental stages. One example is provided by the timely increase of Necdin and DLX transcriptional activators and the decrease of MSX repressors, which promote the progressive rise of *Gnrh1* promoter activity 20, 89, 90. We noticed a slight overlap between biological processes associated with specific trajectories consistent with the idea that each trajectory may shape biological networks and signaling pathways for GnRH neuron development (e.g., T01 - neuronal maturation and connectivity).

Our trajectory analysis can help reveal novel possible biological mechanisms for genes already associated with CHH. For instance, the presence of congenital GnRH deficiency in patients harboring mutations in *TBX3* could be explained by its role in driving the specification of hypothalamic neurons from the *Pomc* lineage producing Kisspeptin 91-93, the most potent activator of GnRH neuron. However, showing high expression in the nasal compartment at early developmental stages, our results suggest that *Tbx3* could modulate GnRH neuron migration during embryonic development. This is consistent with the known role of *Tbx3* in controlling cell migration and invasion by promoting pro-migratory intracellular signaling and modulating the expression of genes involved in cell adhesion 94, 95.

By combining expression trajectories with protein interaction networks, we inferred spatiotemporal dynamics of the cell-to-cell communication between GnRH neurons and their surrounding environment, which we validated with spatial transcriptomic analysis. This approach allowed us to re-discover known signaling modules (e.g., Semaforins/Plexins 54, 56, Netrin/DCC 58-60), to strengthen old candidates (e.g., ephrins 61, Endothelins 62, 63) and highlight new candidate gene pathways such as Neurexins.

Using spatial transcriptomics analysis, we assigned specific signaling modules that emerged from our CCC analysis to distinct stages of GnRH neuron development by spatially resolving more than 100 ligand and receptor genes. Two of these modules involved *Wnt5a* and *Wnt7b,* two members of the Wnt family, a large group of secreted proteins initially known for their role in morphogenesis and patterning during development 96, 97, and later associated with a variety of processes, including brain development, cancer, and stem cell biology 98-100. Wnt signaling modulates the development of the olfactory placode 101, which gives rise to GnRH neurons and olfactory sensory neurons, but also GnRH neuron differentiation 102 and the extension of olfactory sensory neuron projections 103, 104. Interestingly, spatially coordinated action of distinct Wnt proteins plays a crucial role in neuronal guidance. Indeed, opposite gradients of *Wnt5a* and *Wnt7b* have been found to control proper wiring in the developing hindbrain, where dopaminergic axons are repelled by Wnt5a and attracted to Wnt7b 105. Our results show opposite gradients of these two proteins along the migratory route of GnRH neurons, suggesting a role in their guidance. In particular, Wnt5a could act on GnRH neurons migrating in the nose by pushing them toward the brain, while Wnt7b could guide the projections of GnRH neurons that reached the brain by attracting them to the MBH.

Our study reveals an emerging link between GnRH neuron trajectories and the genetic basis of human reproduction. Previous GWAS studies identified many loci associated with the reproductive traits in humans 106-108, including genes affecting fertility via energy homeostasis or directly controlling the activity of GnRH neurons or their activators (i.e., Kisspeptin neurons) 109-111. Here, we show that genes associated with human reproductive onset through GWAS are enriched explicitly in GnRH neuron trajectories with increased expression at late embryonic development. At this stage, GnRH neurons have reached their final destination in the hypothalamus and start integrating into functional circuits by establishing connections with key partners such as kisspeptin neurons 65, 112. In line with this, functional enrichment analysis of these trajectories showed a preferential involvement in the establishment of neuronal projections and synaptic contacts, supporting the idea that variants in these genes could contribute to the timing of puberty by modulating the initiation of GnRH neuron integration into their hypothalamic network. This idea is further corroborated by another study which independently confirmed the association between genes in GnRH neuron trajectories and human reproductive traits using 660 age-at-menarche genes identified after genetic analysis in ~800,000 women from UK Biobank 113.

In contrast, genes involved in CHH have been progressively linked to distinct aspects of GnRH neuron biology, including embryonic development and adult homeostasis, which have been preferentially associated with KS and nCHH, respectively 73, 114-116. Our trajectory-based burden analysis illustrates the complexity of CHH genetic architecture, showing distinct profiles for nCHH and KS subphenotypes. In particular, we found the most significant enrichment of likely pathogenic variants in KS patients and genes up-regulated in GnRH neurons during the early migratory phase.

In summary, our genetic analyses support the idea that the timing of puberty and its absence due to CHH are under the influence of distinct sets of genes operating respectively at late or early stages of GnRH neuron development. This functional segregation is consistent with emerging evidence showing different genetic architectures of congenital delay of puberty (CDGP) and CHH 117, 118, and suggests the potential interest of these genes for molecular diagnosis. Indeed, distinguishing CDGP from CHH in young patients (12-13 years in girls and 13-14 years in boys) is challenging yet critical for an appropriate therapeutic plan as well to reduce anxiety in patients and caregivers 119. Indeed, while CDGP is self-limited and usually does not require treatment, CHH needs intervention for a timely and complete pubertal development 73, 120. However, the low molecular diagnosis rate in CHH patients and the limited number of known genes causing CDGP, represent an obstacle to efficient genetic testing and illustrates the need for novel gene candidates 118, 119.

In conclusion, the comprehensive analysis of gene expression dynamics in GnRH neurons during embryonic development expands our understanding of the underlying molecular mechanisms. We highlighted the importance of GnRH neuron expression trajectories in coordinating crucial biological processes across embryonic development. Finally, we illustrated their links with critical aspects of human reproduction and the diagnostic potential in the genetic discrimination of transient and permanent forms of GnRH deficiency.

## Methods

### Animals

*Gnrh::Gfp* mice, a generous gift of Dr. Daniel J. Spergel (Section of Endocrinology, Department of Medicine, University of Chicago, IL) 16, were housed at room temperature (22°C) with a 12-h-light/12-h-dark cycle and free access to water and food. All experimental protocols were performed following the Swiss animal welfare laws under the authorization of the Service de la consummation et des affaires vétérinaire Vaud. *Gnrh::Gfp* embryos were harvested at E12.5, E14.5, and E18.5 (plug day, E0.5) and used for immunostaining or for the microdissection of nasal and brain regions for GnRH neuron isolation.

### Isolation of GnRH neurons using fluorescence-activated cell sorting

The nose and forebrain microdissections from *Gnrh::Gfp* embryos were enzymatically dissociated using a Papain Dissociation System (Worthington, Lakewood, NJ) to obtain single-cell suspensions 75, 121. Cells were stained with a far-red cell membrane-permeant nuclear dye (RedDot™1, Biotium) to specifically select live cells and DAPI to exclude dead cells. Immediately after staining, FACS was performed on a MOFLO ASTRIOS EQ (BD Bioscience), and, for each microdissection, 500 to 1500 GFP+ and 2000 GFP- cells were sorted directly into 10 μl of extraction buffer.

### RNA processing and transcriptomic profiling

Extraction of total RNA was carried out using Acturus PicoPure™ RNA Isolation Kit (Thermofisher) following the manufacturer's protocol. Concentration and integrity were assessed using the Qubit RNA HS Assay Kit (Life Technologies) and High Sensitivity RNA ScreenTape Assay (Agilent), respectively. Libraries were prepared following the BRB-seq protocol using at least 15 ng total RNA per sample. Sequencing was performed using the Illumina NextSeq 500 platform, and data preprocessing, including sample demultiplexing and alignment, was performed to obtain gene count matrices 122.

### Differential gene expression, functional classification, and enrichment analysis

We performed gene expression analysis on RNAseq data using the edgeR quasi-likelihood method 123. Raw count data were first preprocessed and normalized for differences in library size and composition. Differential expression analysis was then performed using a quasi-likelihood framework, which models both biological variability and technical noise. If not otherwise indicated, genes were considered differentially expressed if they had a false discovery rate (FDR) adjusted p-value below 0.05 and a fold change greater than 2.

Spatiotemporal trajectory classification was performed separately on data for GnRH neurons and GFP^-^ cells. First, differential gene expression analysis was performed in each of the three transitions between the four developmental steps. Then, all transitions were classified according to statistical significance (pval<0.01; fold change > 2), and each gene was annotated with an array combining the results of the three consecutive transitions (Figure S2 and S3).

Computation of gene set overlap for GnRH neuron signature genes has been performed using MSigDB resources (http://www.gsea-msigdb.org/gsea/msigdb/annotate.jsp) 124-126. Gene set enrichment analysis (GSEA) was used with custom gene sets derived from transcriptomic data of GnRH neuron cultures derived from induced pluripotent stem cells (iPSCs) 30-32. The significance of enrichment was evaluated by calculating a normalized enrichment score (NES), and gene sets with an FDR-adjusted p-value < 0.05 were considered significantly enriched. Finally, we used g:GOSt web server (https://biit.cs.ut.ee/gprofiler/gost) 127 for functional enrichment analysis with default and custom datasets.

### Dynamic Cell-to-cell communication network

For cell-to-cell communication analysis, we used CellTalkDB (http://tcm.zju.edu.cn/celltalkdb) 53, a manually curated database of ligand-receptor pairs, to annotate all receptors expressed in GnRH neurons as well as ligand expressed in the environment, and build a ligand-receptor interaction network (Figure 4A). Next, we classified both ligand and receptor genes according to the corresponding trajectories (Figure S2 and S3). Finally, the strength of ligand-receptor interactions was inferred by the expression profiles of ligand-receptor pairs using the scalar product of their normalized expression vectors. We obtained an interaction network representing the dynamic interaction between GnRH neurons and their environment during embryonic development (Figure S4). Finally, functional modules and sub-modules were identified based on their local connectivity (i.e., number and strength of edges).

### Spatial transcriptomic data analysis

We utilized spatial transcriptomic data previously generated by Chen et al. 66 from the Mouse Organogenesis Spatiotemporal Transcriptomic Atlas. H5ad files of mouse embryo sections containing the Gnrh1 transcript were identified and downloaded using the STOmicsDB web interface (https://db.cngb.org/stomics), then imported into the cellxgene_VIP framework (*interactivereport/cellxgene_VIP*) 128. Anatomical structures were manually annotated with the VIP_FreeHandLasso tool, using total gene count and *Col3a1* to visualize local tissue architecture. Gene expression in these annotated regions was calculated and visualized with the VIP_DotPlot, and spatial expression was further analyzed using the VIP_Embedding function.

Receptor-encoding gene expression in individual *Gnrh1*-positive spots, serving as a proxy for GnRH neurons, was illustrated with the VIP_TrackPlot function. The spatial expression relationship of two genes in selected cells was viewed with VIP_DualGenes. To calculate the spatial specificity coefficient for candidate GnRH neuron markers, we used the VIP_MarkerGenes function. For broader spatial expression analysis and visualization of candidate GnRH neuron markers, we used Scanpy 129. Gene modules were computed using scanpy.tl.score_genes, a function that reproduces ModuleScore from Seurat R package 130.

### Tissue preparation

After collection, E14.5 embryos (plug day 0.5) were washed in ice-cold PBS and fixed by immersion in a 4% paraformaldehyde solution in 0.1 M phosphate buffer (pH 7.4) overnight at 4 °C. Embryos were cryopreserved by immersion in 20% sucrose in 0.1 M phosphate-buffered saline (PBS) at 4 °C overnight, embedded in an ice-cold OCT medium (optimal cutting temperature embedding medium, Tissue Tek, Sakura, 4583) and frozen on liquid nitrogen-cooled isopentane. 10-μm-thick coronal sections were obtained using a Leica CM3050S cryostat and stored at -20°C until use. After heat-induced antigen retrieval in 10mM citrate buffer (pH 6), sections were blocked for 1hr at room temperature in 10% normal donkey serum and 0.3% Triton X-100 (Sigma Aldrich, T8787) and incubated overnight at 4 °C with primary antibodies followed by 2 h at room temperature with a cocktail of secondary Alexa Fluor-conjugated antibodies (1:500; Jackson ImmunoResearch Europe Ltd).

### Immunohistochemistry

All primary antibodies used in this study have been previously validated by other groups and include anti-GnRH guinea pig antiserum (1:2000, a generous gift from Dr. Erik Hrabovszky, EH#1018) 131; anti-Cntn1 (10 µg/mL; R&D Systems, AF904) 132; anti-Integrin alpha V (1:200; Novus Biologicals, NBP1-85746) 133; Anti-CXCR7 (10 µg/mL; Novus Biologicals, NBP2-24779) 134; anti-Plexin A1 (10 µg/mL; R&D Systems, AF4309) 135; anti-Ecel1 (1:150; Sigma-Aldrich, HPA077424).

Images were acquired using the Leica Thunder Imaging System (x40 objective, 0.6 µm z-scan, 9-11 optical slices). Images for illustration were finally exported in.tiff. and processed (i.e., adjust brightness and contrast and merge images) using Adobe Photoshop (Adobe Systems, San Jose, CA).

### Next-generation sequencing of CHH patients

The CHH cohort included 226 KS and 199 normosmic CHH (nCHH) for a total of 425 unrelated probands (320 males and 105 females) with a majority of European descent. All subjects provided written informed consent, and their clinical phenotype was assessed as previously described 136.

After DNA extraction, paired-end whole exome sequencing was performed at the Denmark facility of BGI (Beijing Genomics Institute) Global (n=106) or at Health 2030 Genome Center (a portion of subjects between 2019 and 2021, n=124), while whole genome sequencing (n=195) was performed using DNBSEQ technology through the Denmark facility of BGI (Beijing Genomics Institute) Global 137. Briefly, the resulting raw sequences (fastq files) are processed by an in-house bioinformatics analysis workflow that relies on Sentieon DNASeq, a GATK-compliant toolbox that maps the reads to the human reference sequence (GRCh37) and detects variants 138, 139. Identified variants are then annotated with minor allele frequencies (MAFs) from gnomAD (v2.1.1, n=54'704 controls) (http://gnomad.broadinstitute.org) and with multiple pathogenicity prediction tools 140, 141 using ANNOVAR 142. The GnomAD exomes vcf file was downloaded and annotated with ANNOVAR using the same databases to ensure coherence.

For the genetics burden, we applied a series of filters to minimize false positive calls and sequencing artifacts while preserving truly positive calls. This strategy, detailed below, was used for both the CHH cohort and the GnomAD controls whenever possible. We excluded variants with a popmax frequency in GnomAD higher than 0.01%, given the prevalence of CHH 73. We retained nonsense variants (i.e., stop gain, frameshift, acceptor-donor splice sites ± 2bp from an exon or SpliceAI 143) that passed strict filters guided by GATK recommendations (minimum quality score of 50, mapping quality > 55 and Mapping Quality Rank Sum Test > -2.5) [https://gatk.broadinstitute.org/hc/en-us/articles/360035890471-Hard-filtering-germline-short-variants]. Variants with an allelic depth ratio under 20% or located in segmental duplications 144 were discarded. Putative private or ultra-rare variants that were frequent (> 3 times) in a local genetic database of healthy individuals (n=300) were also removed, as they were considered systematic sequencing artifacts. Furthermore, variants that were present in excess of pedigrees (>5) or flagged in GnomAD, as well as indels involving more than three nucleotides, were excluded due to the higher error rate in calls, primarily from alignment issues. After applying all the filters, the subjects with at least one variant in the same trajectory were counted. Each variant in GnomAD's data was assumed to come from a different individual, with a ceiling at the number of controls, and a contingency table with affected and wild-type alleles was constructed. A one-sided Fisher's Exact test for each trajectory was then performed to estimate the enrichment of variant alleles in the CHH cohort vs. GnomAD controls. This analysis was applied to synonymous variants through the same filters to control for the overall inflation of numbers from potential unaccounted-for sources.^70^ From the odds ratio obtained, a 95% confidence interval for the odds ratios could be estimated as follows: 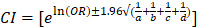 (1)with a,b,c and d being the count values from the contingency table. Using (1), it is then possible to calculate a z-score statistic between the estimated odds ratios from PTVs and synonymous variants following:
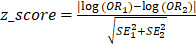
 (2)with 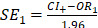 . Only the trajectories passing a Bonferroni correction were retained and considered as having a significant enrichment of PTVs relative to synonymous (i.e., the loss-of-function burden test has a significantly higher odds ratio than the synonymous burden testing CHH). Finally, this process was repeated separately for nCHH and KS probands to evaluate phenotype specificity.

## Supplementary Material

Supplementary figures and tables.
