# Supplementary material for: Transcriptomic profiling of murine GnRH neurons reveals developmental trajectories linked to human reproduction and infertility
Source: Theranostics. 2025 Feb 26;15(8):3673–92. doi: 10.7150/thno.91873 (PMC11905127; doi:10.7150/thno.91873)
Supplement: Supplementary file 1 — Supplementary figures and tables. [file thnov15p3673s1.zip › Supplementary Figures.pdf]

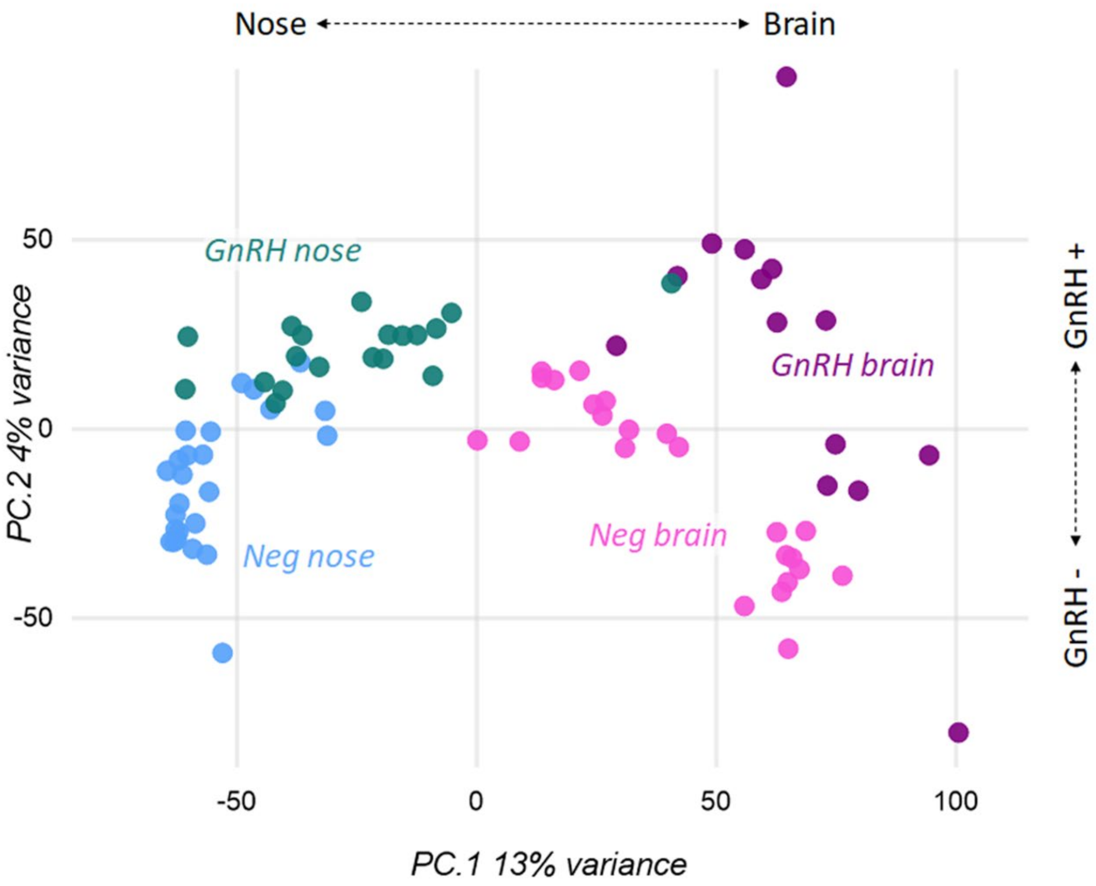

Supplementary Figure 1 | **Principal Component Analysis (PCA) of RNA sequencing data from GnRH^{+} and GnRH^{-} mouse embryonic cells.**

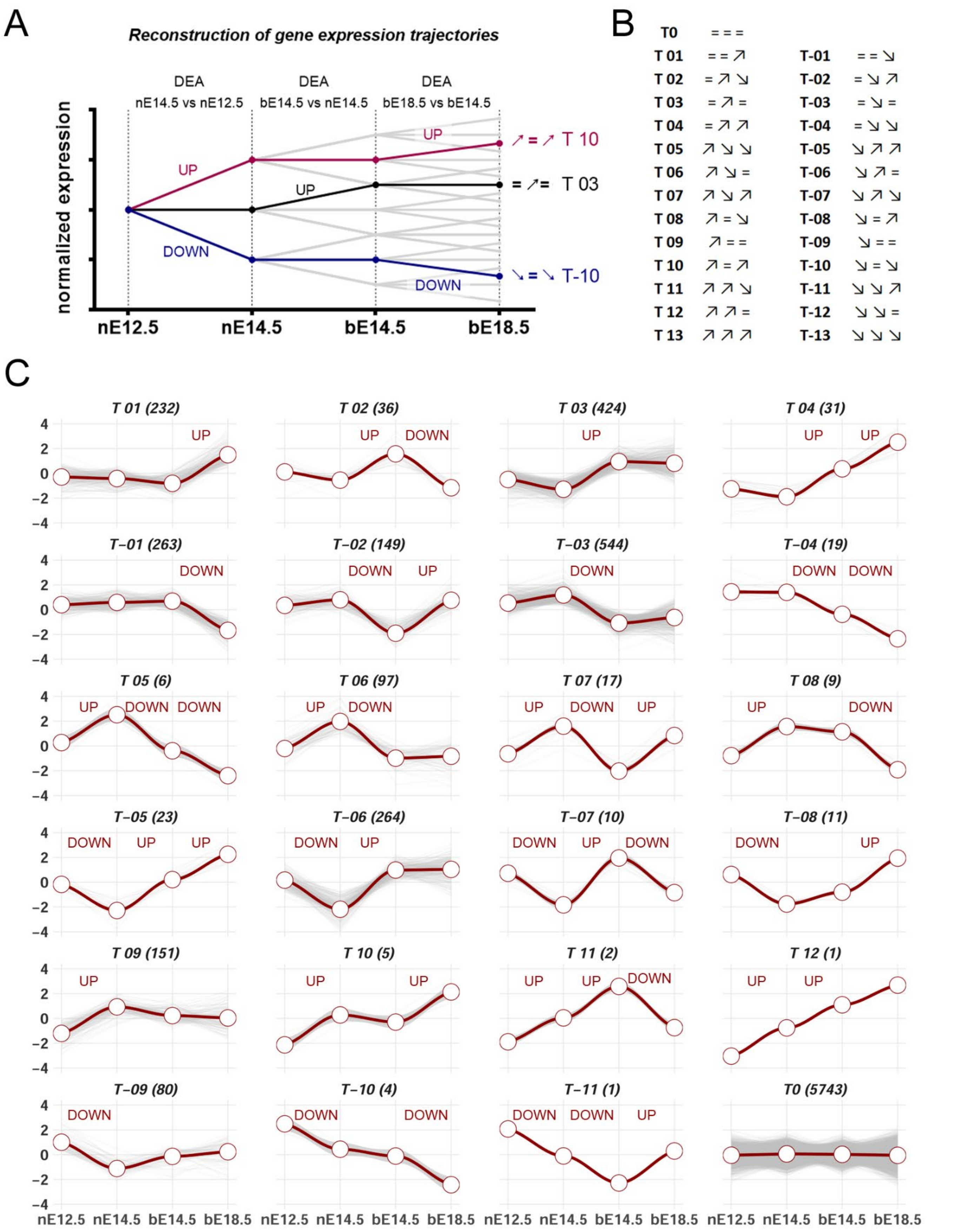

Supplementary Figure 2 | **GnRH+ cells gene expression dynamics**. (A) Algorithm to assign trajectory names to expression dynamics according to significant expression changes across consecutive developmental stages (UP: Log2FoldCchange > 1 and p value < 0.01; DOWN: Log2FoldCchange < -1 and p value < 0.01). (B) Pattern description of all possible trajectories in the GnRH transcriptomics dataset. (C) Expression of all detected genes in GnRH+ cells organized by trajectory. Grey lines represent individual genes and average gene expression is shown in red.

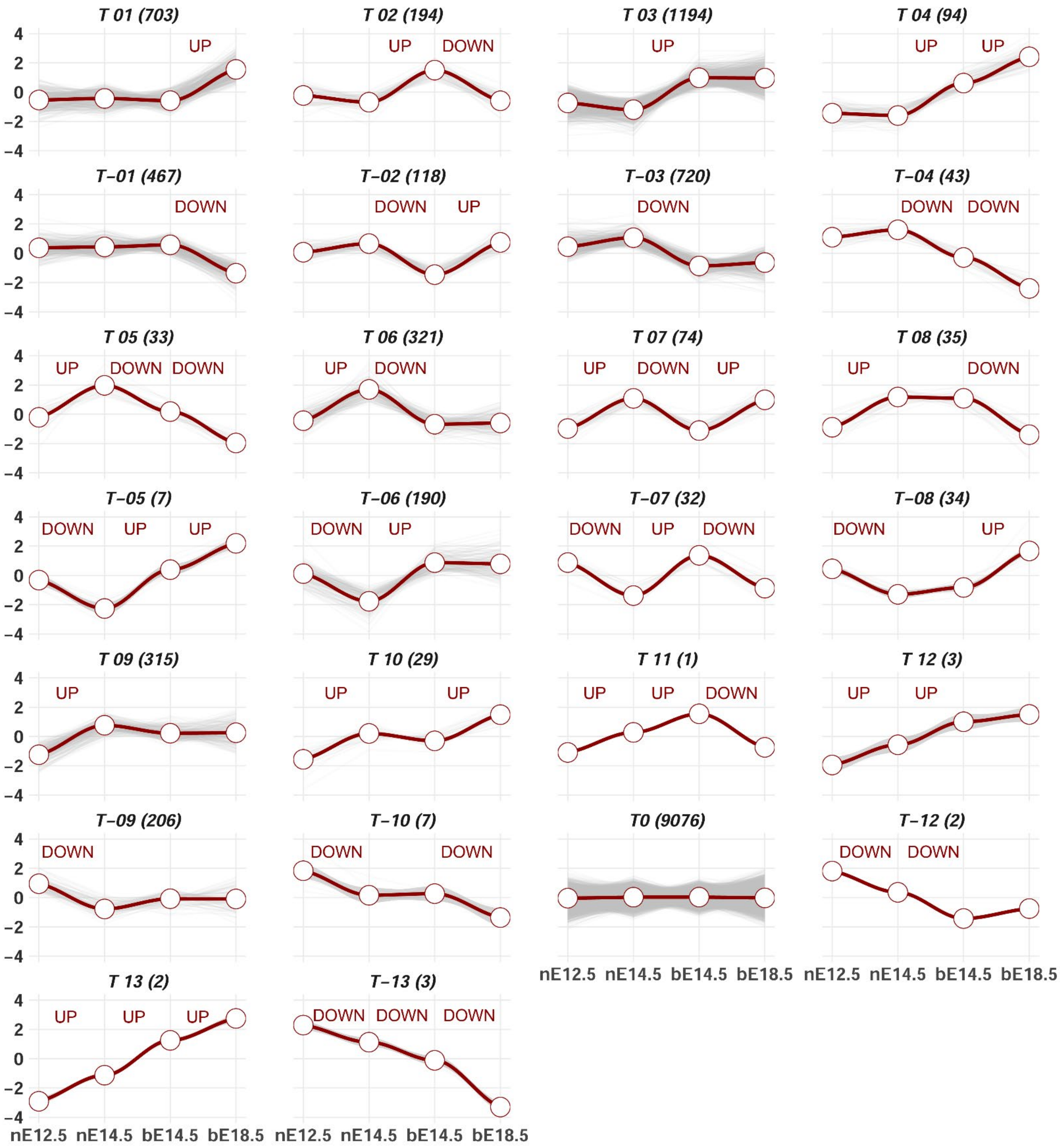

Supplementary Figure 3 | **GnRH⁻ cells gene expression dynamics**. Expression of all detected genes in GnRH- cells organized by trajectory. Average gene expression is shown in red while grey lines represent individual genes.

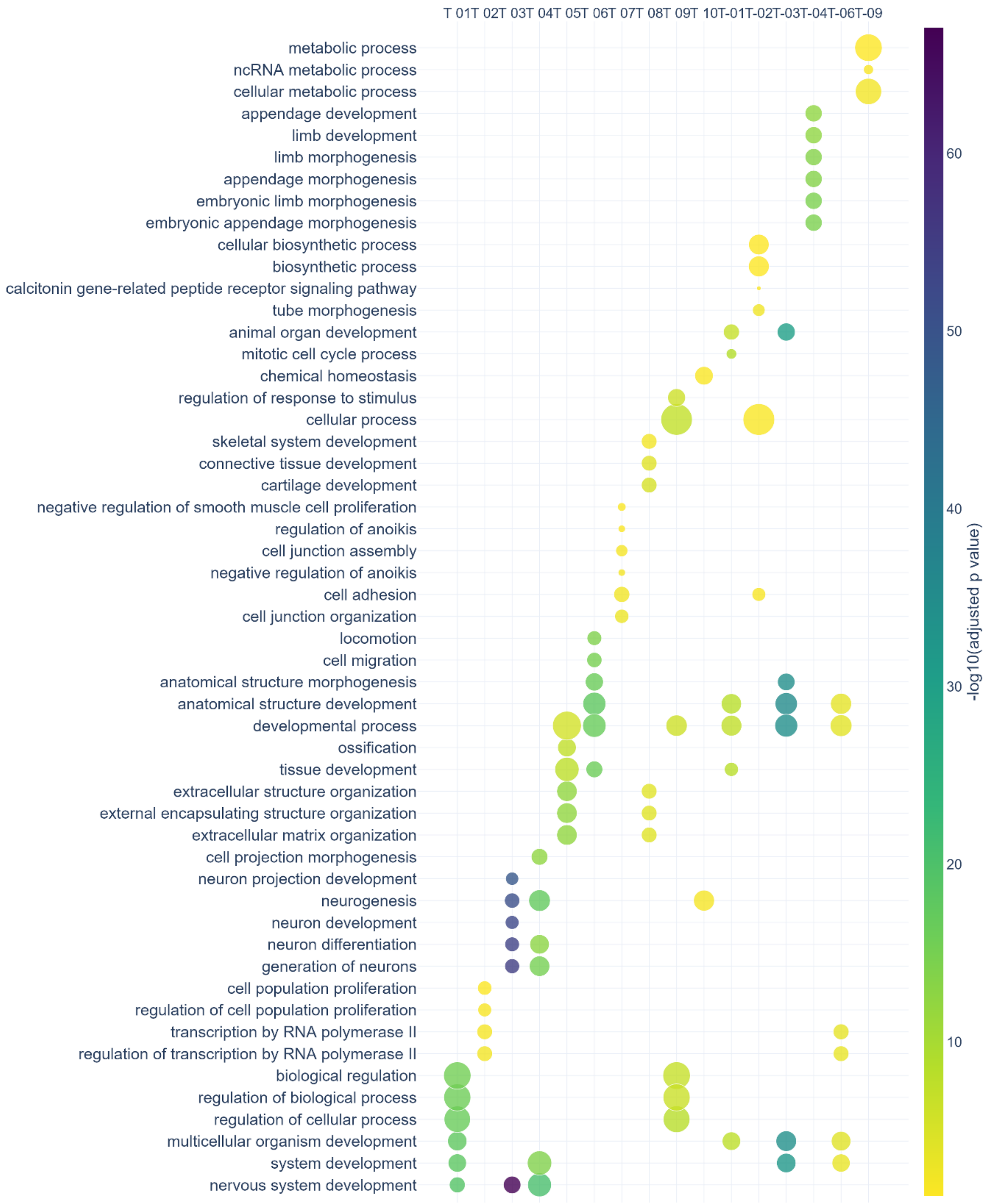

Supplementary Figure 4 | **Functional enrichment analysis in GnRH^{-} cells**. Scatter plot showing the top six significant terms in the main trajectories of GnRH- cells after functional enrichment analysis within biological processes from gene ontology database.

Top candidate genes in GnRH neuron trajectories

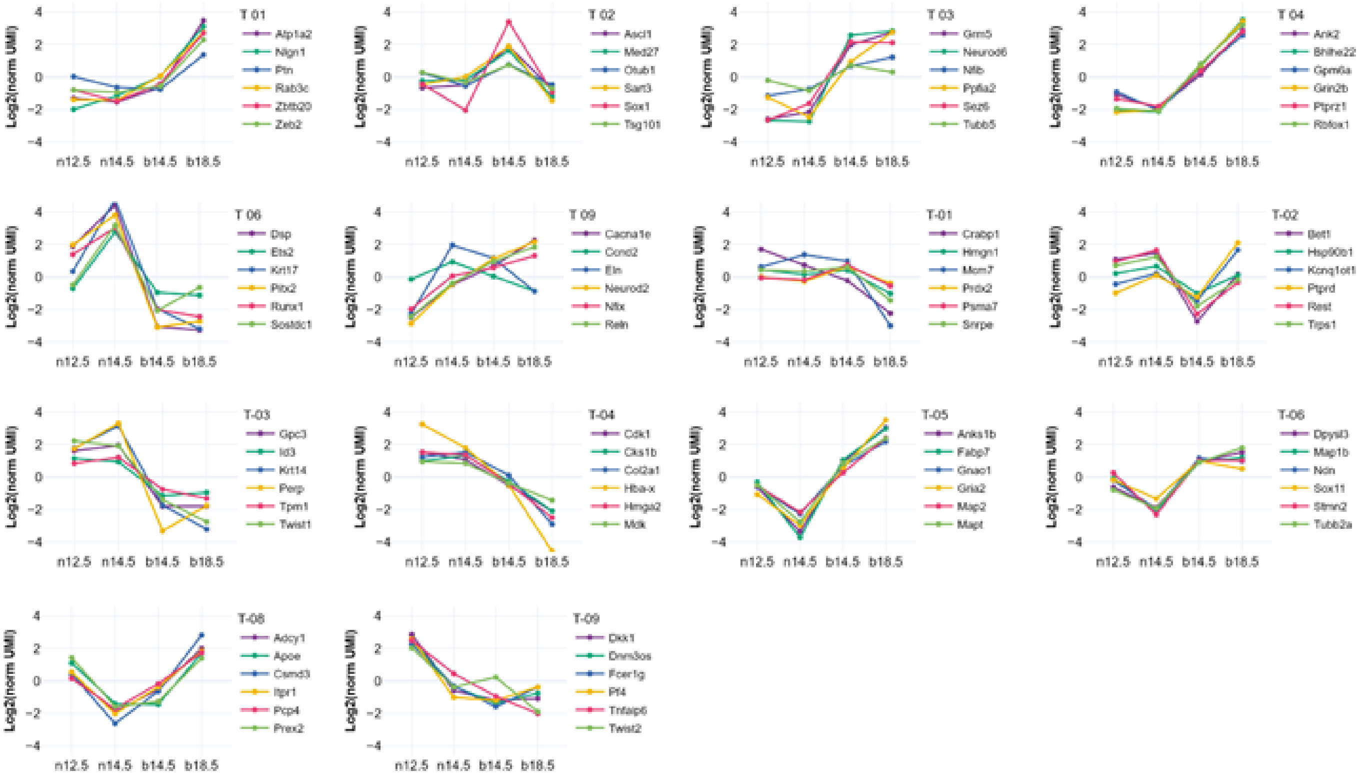

Top candidate genes in GnRH negative cell trajectories

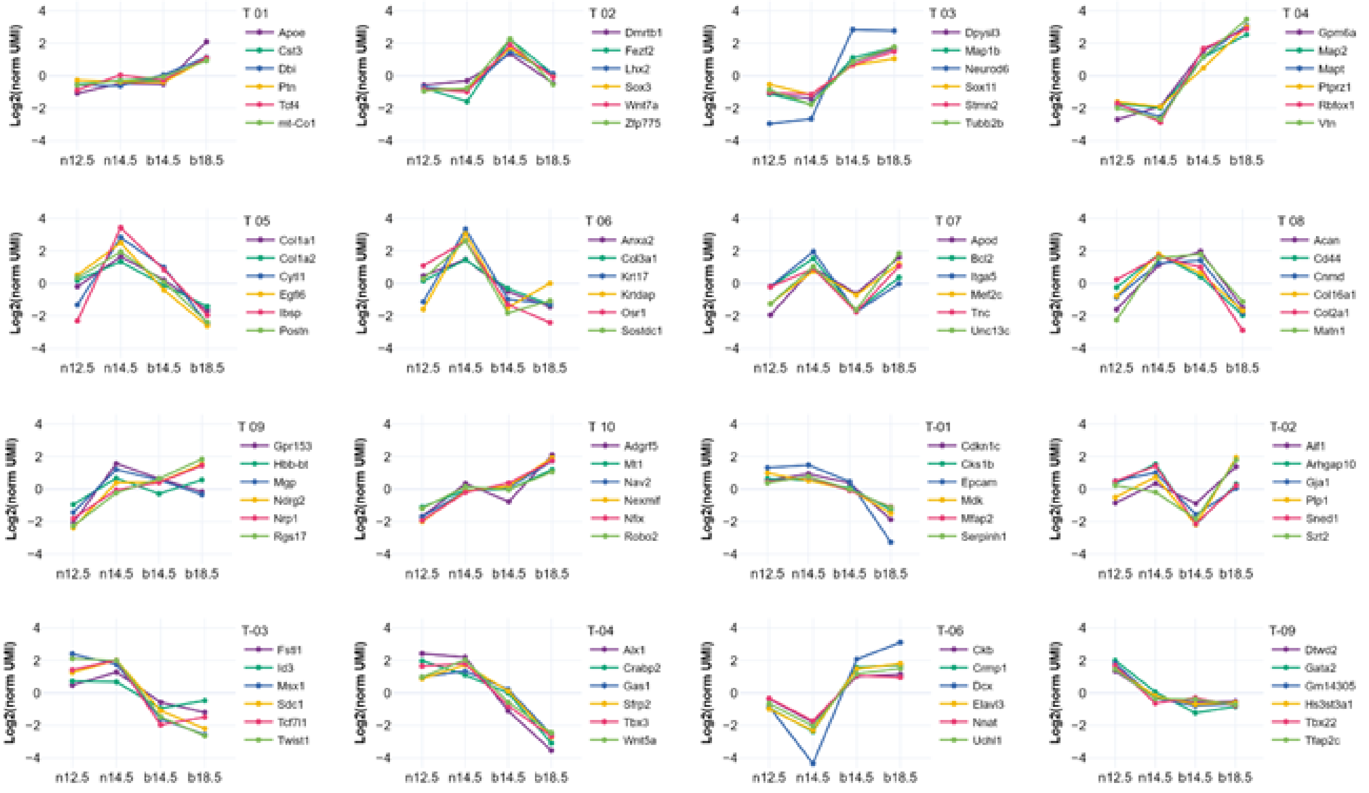

Supplementary Figure 5 | **Expression profiles of top genes from trajectories enriched in biological processes**. Normalized expression profiles and trajectory classification of top genes emerging from representative trajectories significant after functional enrichment analysis in both $GnRH^+$ and $GnRH^-$ cells.

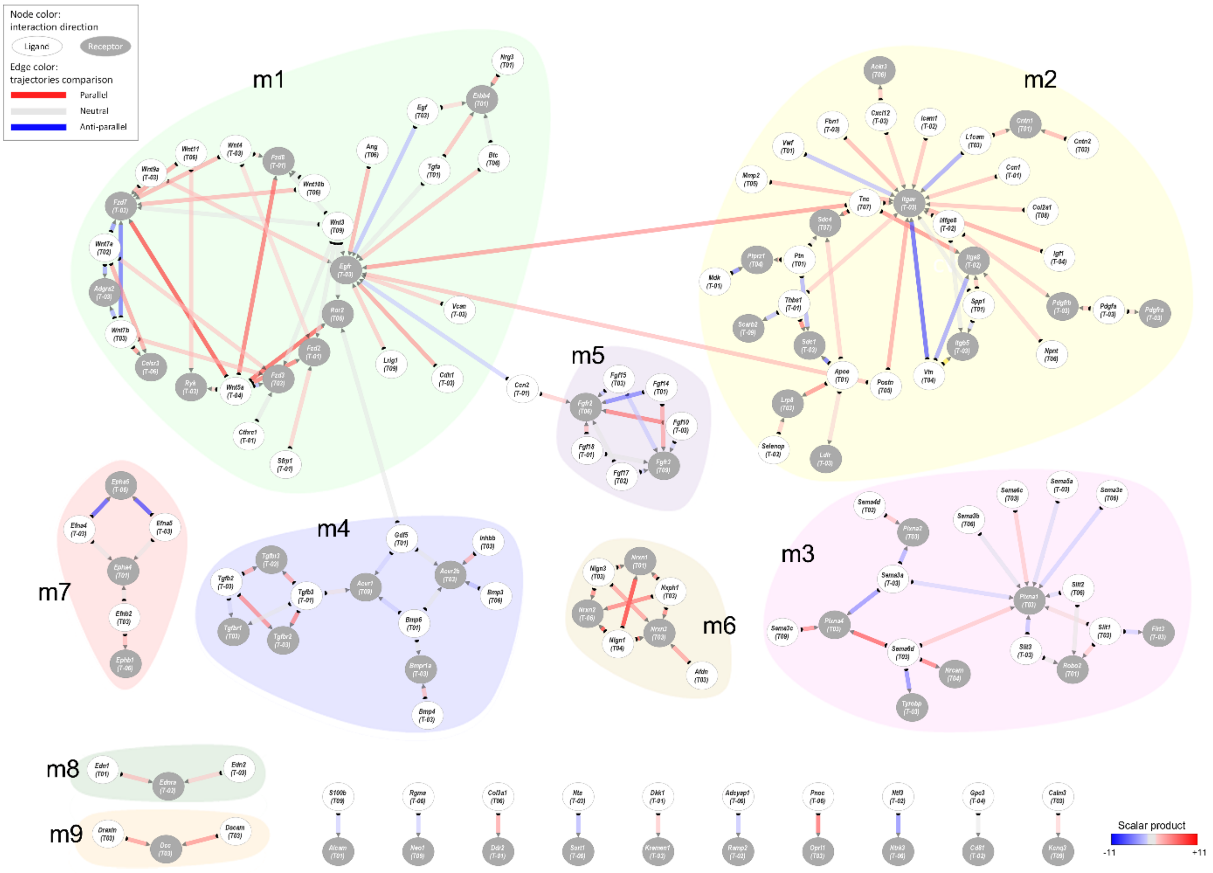

Supplementary Figure 6 | **Full dynamic cell-to-cell communication networks in GnRH neuron development.** PPI network and gene expression profiles of different modules of ligands and their respective receptors (T0 nodes are hidden for visual clarity). Node color discriminates between GnRH neurons (grey) receptors and ligands expressed in the environment (white). The degree of coordinated expression (i.e., scalar product) is color-coded on edges.

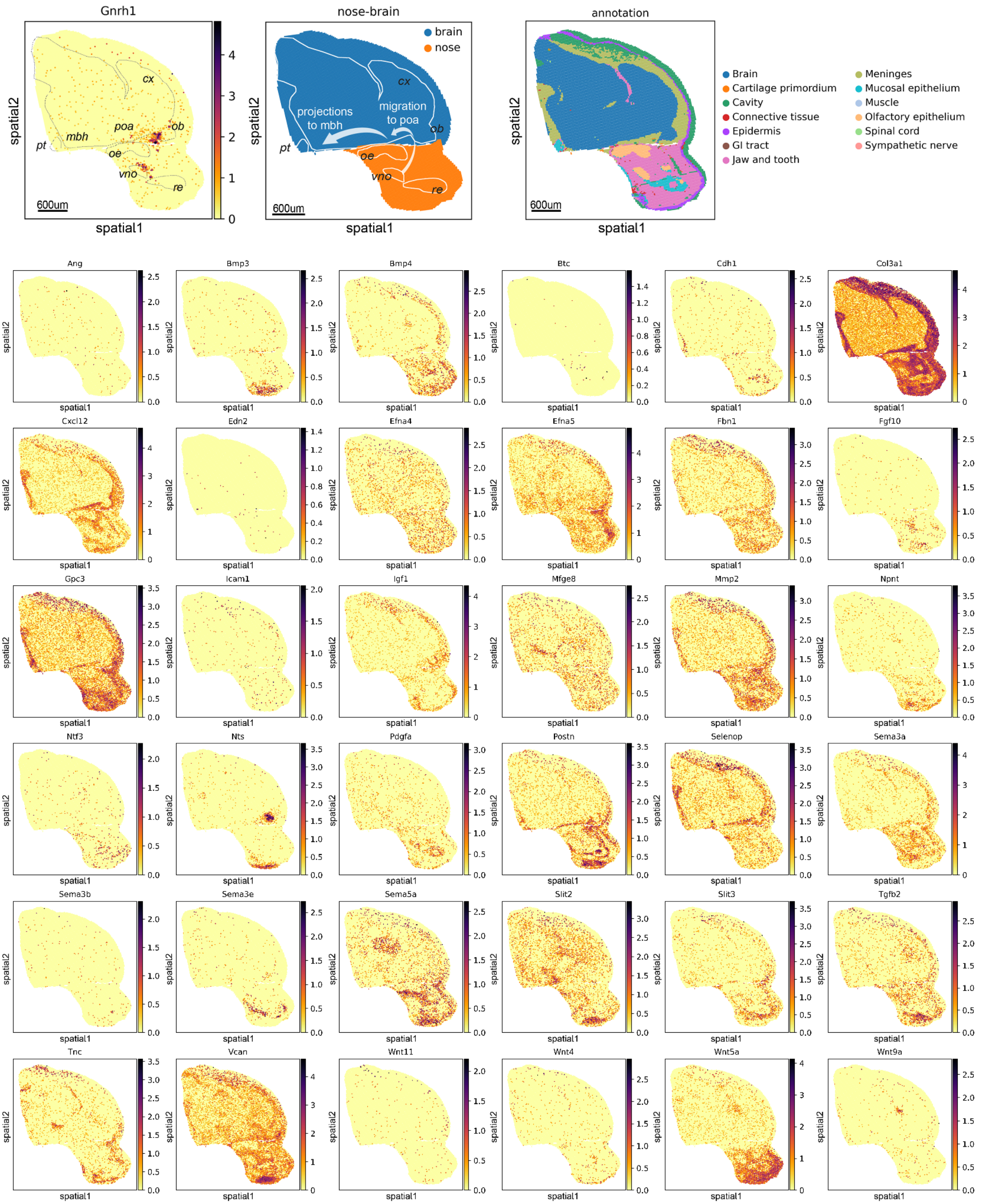

Figure 7 | **Spatial transcriptomics validation of CCC-ligand genes with preferential expression in the nasal region during GnRH neuron development.**

Embedding plots showing spatial visualization of transcriptomic data reanalyzed from a mouse embryo section at E13.5 (MOSTA). Annotation of anatomical regions relevant for GnRH neuron development followed by spatial visualization transcript encoding CCC-ligans predicted to be preferentially expressed in the nasal region. expression Spot size 25um. *Cx, cortex; pt, pituitary; mbh, mediobasal hypothalamus; poa, preoptic*

area of the hypothalamus; ob, olfactory bulb; oe, olfactory epithelium; vno, vomeronasal organ; re, respiratory epithelium. T, trajectory; SP, spatial pattern.

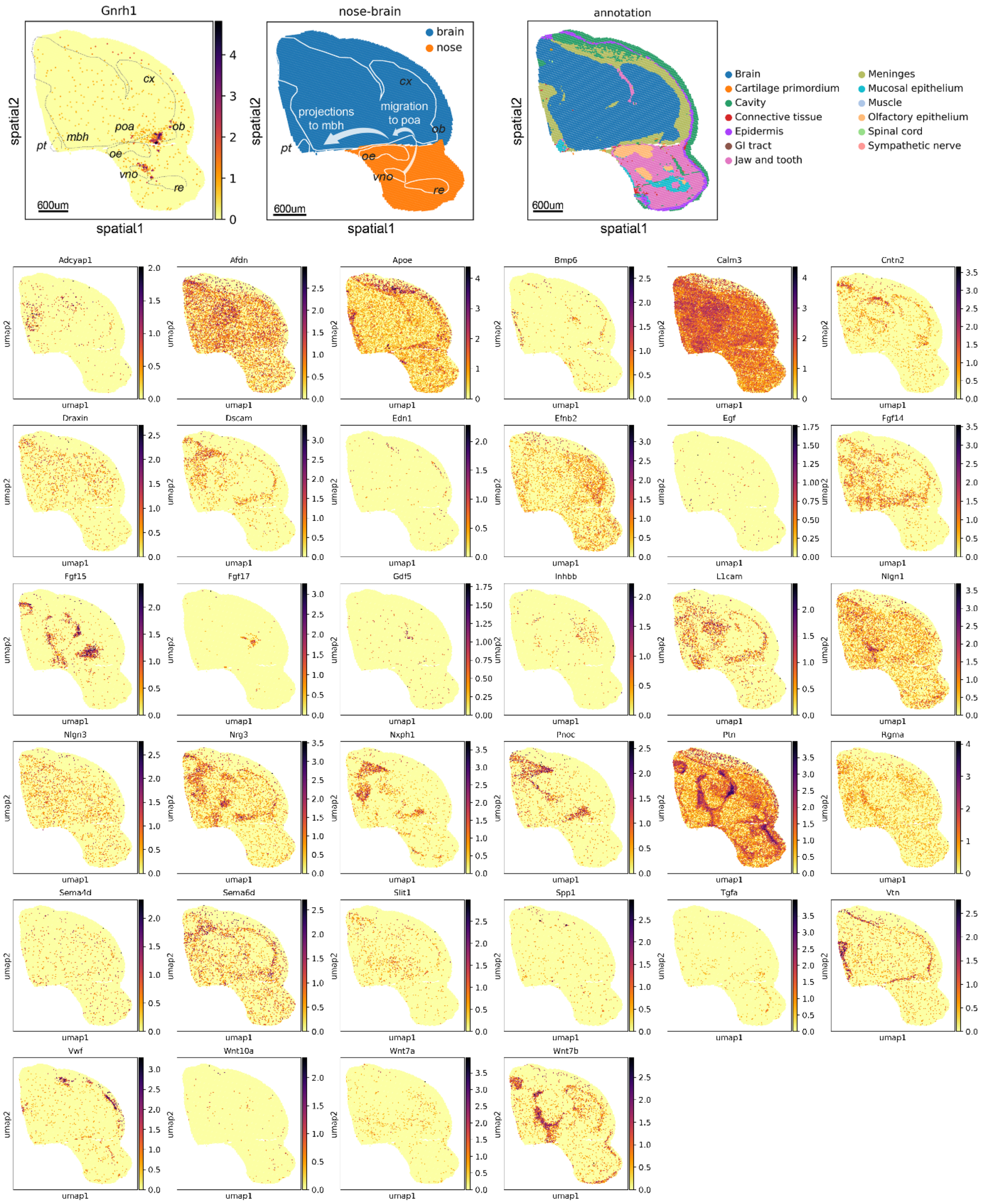

Figure 8 | **Spatial transcriptomics validation of CCC-ligand genes with preferential expression in the forebrain region during GnRH neuron development.**

Embedding plots showing spatial visualization of transcriptomic data reanalyzed from a mouse embryo section at E13.5 (MOSTA). Annotation of anatomical regions relevant for GnRH neuron development followed by spatial visualization transcript encoding CCC-ligans predicted to be preferentially expressed in the forebrain region. expression Spot size 25um. *Cx, cortex; pt, pituitary; mbh, mediobasal hypothalamus; poa, preoptic area of the hypothalamus; ob, olfactory bulb; oe, olfactory epithelium; vno, vomeronasal organ; re, respiratory epithelium*. T, trajectory; SP, spatial pattern.

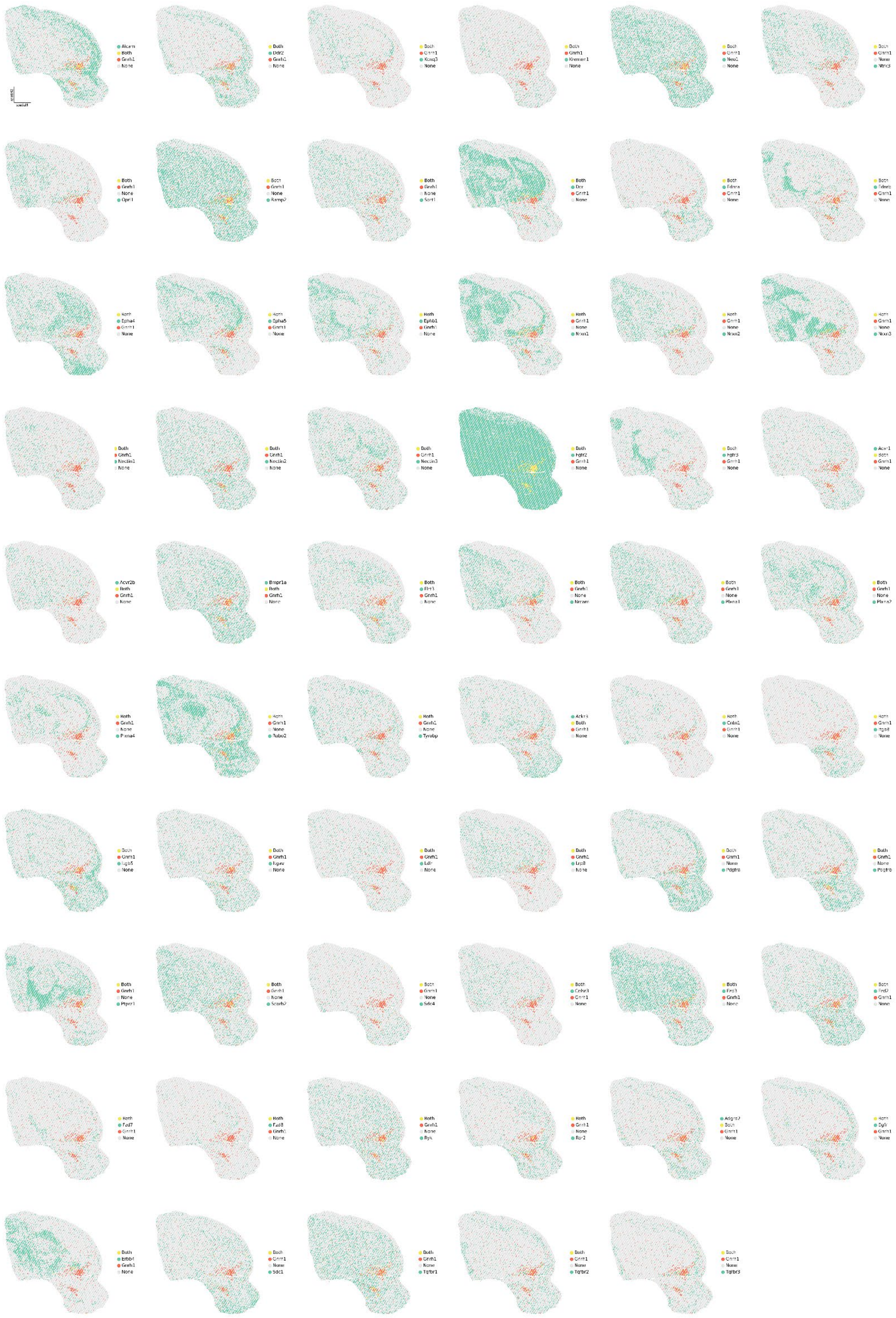

Alcam Both Gnrh1 None
Both Ddr2 Gnrh1 None
Both Gnrh1 Kcnq3 None
Both Gnrh1 Kremen1 None
Both Gnrh1 Neo1 None
Both Gnrh1 None Ntrk3
Both Gnrh1 None Oprl1
Both Gnrh1 None Ramp2
Both Gnrh1 None Sort1
Both Dcc Gnrh1 None
Both Ednra Gnrh1 None
Both Ednrb Gnrh1 None
Both Epha4 Gnrh1 None
Both Epha5 Gnrh1 None
Both Ephb1 Gnrh1 None
Both Gnrh1 None Nrxn1
Both Gnrh1 None Nrxn2
Both Gnrh1 None Nrxn3
Both Gnrh1 Nectin1 None
Both Gnrh1 Nectin2 None
Both Gnrh1 Nectin3 None
Both Fgfr2 Gnrh1 None
Both Fgfr3 Gnrh1 None
Acvr1 Both Gnrh1 None
Acvr2b Both Gnrh1 None
Bmpr1a Both Gnrh1 None
Both Flrt3 Gnrh1 None
Both Gnrh1 None Nrcam
Both Gnrh1 None Plxna1
Both Gnrh1 None Plxna2
Both Gnrh1 None Plxna4
Both Gnrh1 None Robo2
Both Gnrh1 None Tyrobp
Ackr3 Both Gnrh1 None
Both Cntn1 Gnrh1 None
Both Gnrh1 Itga8 None
Both Gnrh1 Itgb5 None
Both Gnrh1 Itgav None
Both Gnrh1 Ldlr None
Both Gnrh1 Lrp8 None
Both Gnrh1 None Pdgfra
Both Gnrh1 None Pdgfrb
Both Gnrh1 None Ptprz1
Both Gnrh1 None Scarb2
Both Gnrh1 None Sdc4
Both Celsr3 Gnrh1 None
Both Fzd3 Gnrh1 None
Both Fzd2 Gnrh1 None
Both Fzd7 Gnrh1 None
Both Fzd8 Gnrh1 None
Both Gnrh1 None Ryk
Both Gnrh1 None Ror2
Adgra2 Both Gnrh1 None
Both Egfr Gnrh1 None
Both Erbb4 Gnrh1 None
Both Gnrh1 None Sdc1
Both Gnrh1 None Tgfbr1
Both Gnrh1 None Tgfbr2
Both Gnrh1 None Tgfbr3

Figure 9 | **Spatial transcriptomics co-localisation analysis of *Gnrh1* transcript with genes encoding CCC-receptors during GnRH neuron development.**

Embedding plots showing spatial visualization of transcriptomic data reanalyzed from a mouse embryo section at E13.5 (MOSTA). Spot size 25um.

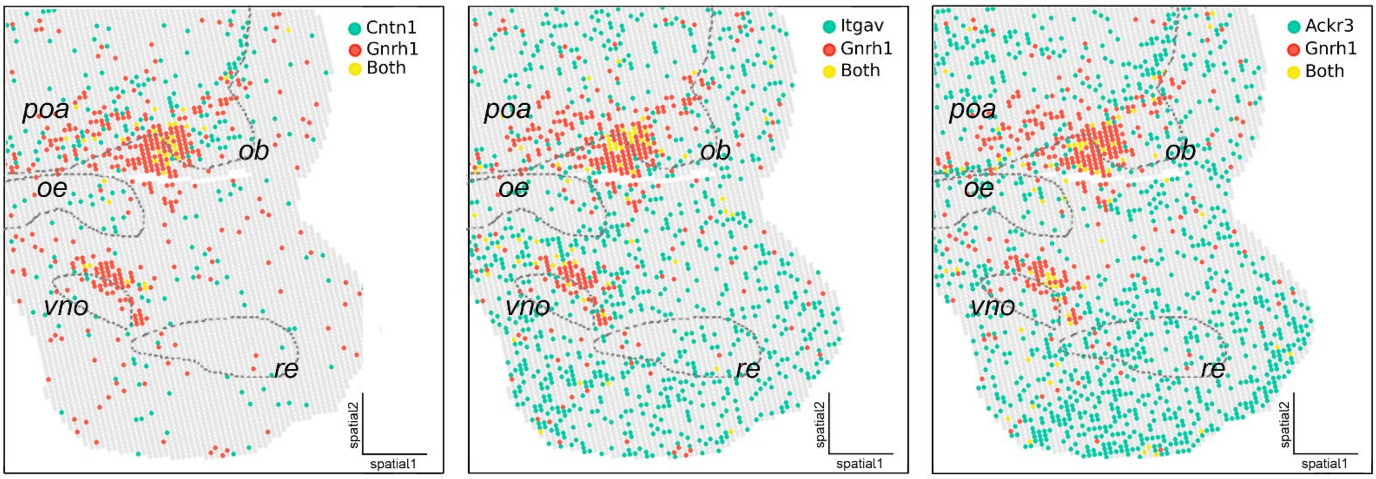

Figure 10 | **Heterogeneous co-localisation of *Cntn1*, *Itgav* and *Ackr3* with *Gnrh1* transcript during GnRH neuron development.**

Embedding plots illustrating heterogeneous co-localization of Cntn1, Itgav and Ackr3 with Gnrh1 in the context of relevant anatomical regions (gray dotted lines). Spot size 25um. 1090 Cx, cortex; pt, pituitary; mbh, mediobasal hypothalamus; poa, preoptic area of the hypothalamus; 1091 ob, olfactory bulb; oe, olfactory epithelium; vno, vomeronasal organ; re, respiratory epithelium. T, 1092 trajectory; SP, spatial pattern.

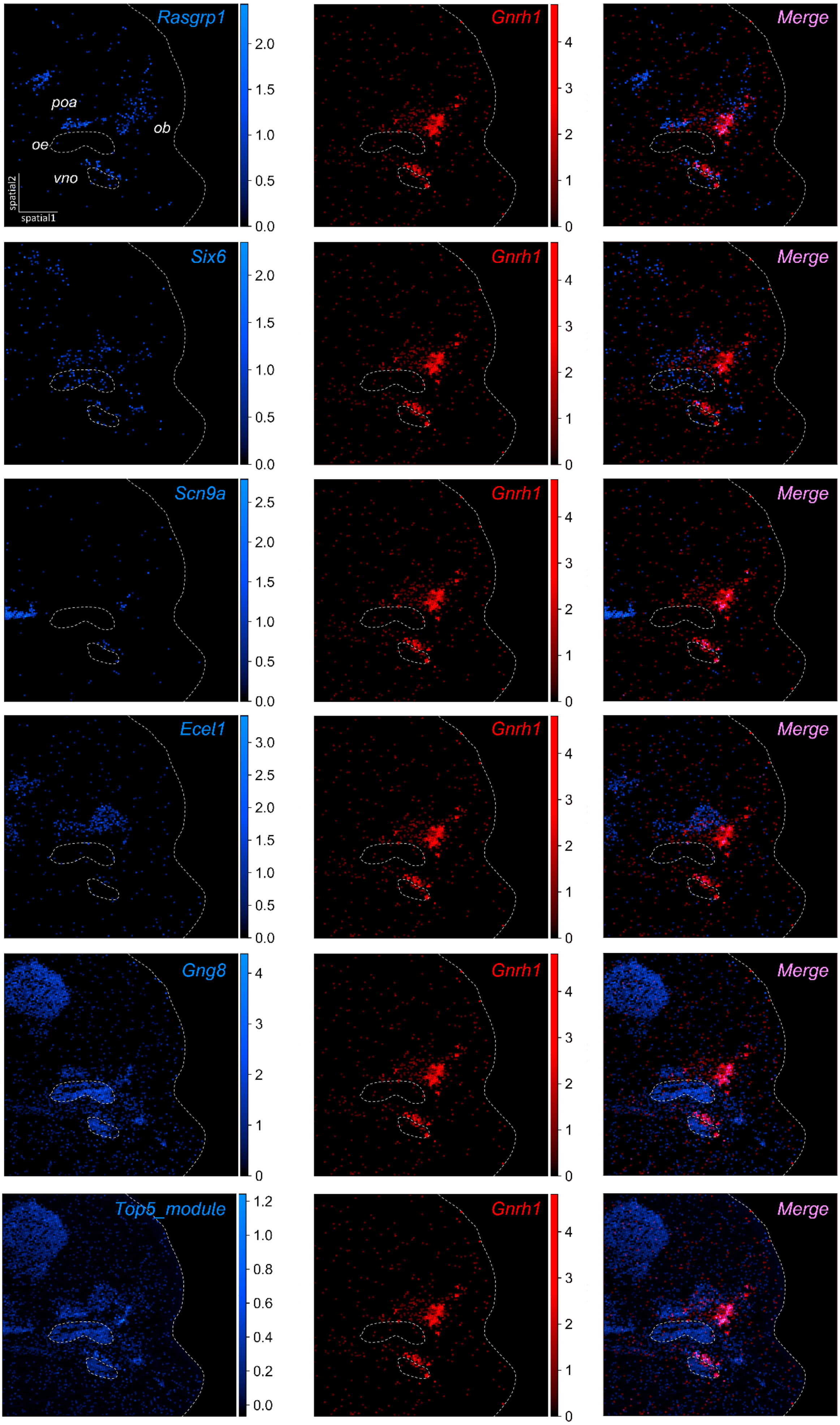
Rasgrp1
poa
ob
oe
vno
spatial2
spatial1
Six6
Scn9a
Ecel1
Gng8
Top5_module
Gnrh1
Merge

Figure 11 | **Spatial transcriptomics analysis of candidate markers for GnRH neurons.**

Embedding plots of transcriptomic data reanalyzed from a mouse embryo section at E13.5 (MOSTA) co-localisation of the top 5 GnRH neuron candidate marker genes with *Gnrh1* transcript. Colored scalebars represent relative gene expression intensity. Section border and relevant anatomical regions are outlined (light gray dotted lines). Spot size 25um. *poa, preoptic area of the hypothalamus; ob, olfactory bulb; oe, olfactory epithelium; vno, vomeronasal organ*.

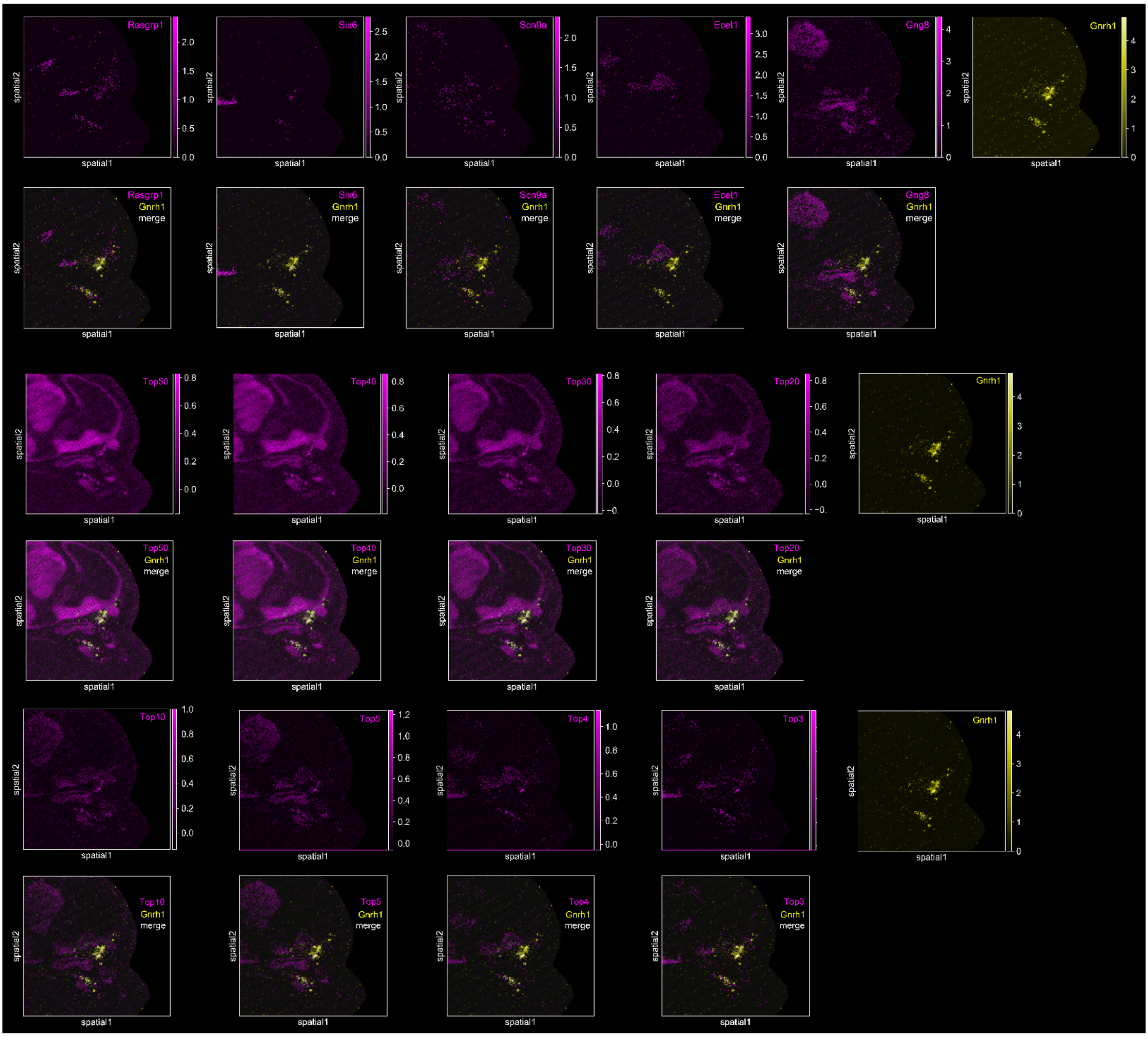

Figure 12 | **Spatial transcriptomics analysis of combined markers for GnRH neurons.**
Embedding plots showing spatial visualization of transcriptomic data reanalyzed from a mouse embryo section at E13.5 (MOSTA). Spot size 25um.
